# MicroRNA Regulation of Cbx7 Mediates a Switch of Polycomb Orthologs during ESC Differentiation

**DOI:** 10.1016/j.stem.2011.12.004

**Published:** 2012-01-06

**Authors:** Ana O'Loghlen, Ana M. Muñoz-Cabello, Alexandre Gaspar-Maia, Hsan-Au Wu, Ana Banito, Natalia Kunowska, Tomas Racek, Helen N. Pemberton, Patrizia Beolchi, Fabrice Lavial, Osamu Masui, Michiel Vermeulen, Thomas Carroll, Johannes Graumann, Edith Heard, Niall Dillon, Veronique Azuara, Ambrosius P. Snijders, Gordon Peters, Emily Bernstein, Jesus Gil

**Affiliations:** 1 Cell Proliferation Group, MRC Clinical Sciences Centre, Imperial College London, Hammersmith Campus, London W12 0NN, UK; 2 Epigenetics Section, MRC Clinical Sciences Centre, Imperial College London, Hammersmith Campus, London W12 0NN, UK; 3 Gene Regulation and Chromatin Group, MRC Clinical Sciences Centre, Imperial College London, Hammersmith Campus, London W12 0NN, UK; 4 Biomolecular Mass Spectrometry and Proteomics Laboratory, MRC Clinical Sciences Centre, Imperial College London, Hammersmith Campus, London W12 0NN, UK; 5 Department of Oncological Sciences, Mount Sinai School of Medicine, 1425 Madison Avenue, New York, NY 10029, USA; 6 Molecular Oncology Laboratory, CRUK London Research Institute, London WC2A 3PX, UK; 7 Institute of Reproductive and Developmental Biology, Imperial College, London W12 0NN, UK; 8 Institut Curie, 26 rue d'Ulm, Paris F-75248, France; 9 Department of Proteomics and Signal Transduction, Max Planck Institute of Biochemistry, 82152 Martinsried, Germany; 10 Department of Molecular Cancer Research, University Medical Center Utrecht, The Netherlands; 11 Weill Cornell Medical College in Qatar, Qatar Foundation, Education City, Doha, Qatar

## Abstract

The Polycomb Group (PcG) of chromatin modifiers regulates pluripotency and differentiation. Mammalian genomes encode multiple homologs of the Polycomb repressive complex 1 (PRC1) components, including five orthologs of the *Drosophila* Polycomb protein (Cbx2, Cbx4, Cbx6, Cbx7, and Cbx8). We have identified Cbx7 as the primary Polycomb ortholog of PRC1 complexes in embryonic stem cells (ESCs). The expression of Cbx7 is downregulated during ESC differentiation, preceding the upregulation of Cbx2, Cbx4, and Cbx8, which are directly repressed by Cbx7. Ectopic expression of Cbx7 inhibits differentiation and X chromosome inactivation and enhances ESC self-renewal. Conversely, Cbx7 knockdown induces differentiation and derepresses lineage-specific markers. In a functional screen, we identified the miR-125 and miR-181 families as regulators of Cbx7 that are induced during ESC differentiation. Ectopic expression of these miRNAs accelerates ESC differentiation via regulation of Cbx7. These observations establish a critical role for Cbx7 and its regulatory miRNAs in determining pluripotency.

## Graphical abstract

**Graphical Abstract:**

**Figure:**
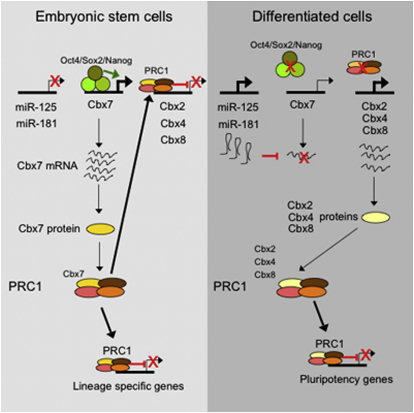

**Highlights:**

► Cbx7 is the primary Pc ortholog of the PRC1 complex in pluripotent cells ► Cbx7 repress its homologs to regulate PRC1 composition during ESC differentiation ► Cbx7 promotes a stem-cell-like state by repressing differentiation ► miR-181 and miR-125 regulate Cbx7 expression during ESC differentiation

## Introduction

The Polycomb Group (PcG) family of transcriptional repressors plays important roles in the epigenetic regulation of pluripotency, differentiation, X chromosome inactivation, and senescence (Sparmann and van Lohuizen, 2006). PcG proteins operate in multicomponent complexes, the best characterized of which are termed Polycomb repressive complexes 1 (PRC1) and 2 (Morey and Helin, 2010). PRC2 catalyzes the trimethylation of histone H3 on lysine 27 (H3K27me3), leading to recruitment of PRC1, which “reads” H3K27me3 and ubiquitylates H2AK119 (Simon and Kingston, 2009). In *Drosophila*, PRC1 is composed of stoichiometric amounts of Polycomb (Pc), Posterior sex combs (Psc), Polyhomeotic (Ph), and Sex combs extra (Sce) proteins; however, because mammalian genomes encode five Pc, six Psc, three Ph, and two Sce orthologs, there are many possible combinations of PRC1 components (summarized in Figure 2A) (Whitcomb et al., 2007).

The reasons for such PcG diversification and the interplay between the different orthologs remain unclear. Biochemical analyses suggest that PRC1 complexes contain a single representative of each subunit, and chromatin immunoprecipitation (ChIP) studies in human fibroblasts have revealed that multiple Pc and Psc orthologs, and therefore multiple PRC1 complexes, can bind simultaneously at the *INK4a/ARF* locus (Maertens et al., 2009). While the two Pc orthologs Cbx7 and Cbx8 are both capable of repressing the *INK4a/ARF* locus in this cell model (Dietrich et al., 2007; Gil et al., 2004), the role of each subunit is also likely to be context dependent, reflecting the combinations expressed in different cell lineages. For example, the striking phenotype of *Bmi1*^−/−^ mice, which have hematological and neurological defects due to a failure in stem cell self-renewal (Sparmann and van Lohuizen, 2006), implies that the other members of the Psc family are unable to compensate for the absence of Bmi1.

Genome-wide ChIP analyses in human and mouse embryonic stem cells (ESCs) have shown that PRC1 and PRC2 localize to the promoters of a subset of genes encoding transcription factors (TFs) required for lineage specification (Boyer et al., 2006; Lee et al., 2006). These promoters contain binding sites for pluripotency-associated TFs, such as Oct4, Sox2, Nanog, and Sall4 (Boyer et al., 2005, 2006; Lee et al., 2006), and are enriched for both histone H3K4me3 and H3K27me3 marks (Azuara et al., 2006; Bernstein et al., 2006a). This so-called bivalent mark enables pluripotent cells to respond rapidly to differentiation signals, and ESCs that lack critical PRC1 or PRC2 components display global derepression of these target genes (Azuara et al., 2006; Boyer et al., 2006). As a result, these ESCs are unstable and more prone to spontaneous differentiation in culture (Boyer et al., 2006), but the simultaneous ablation of PRC1 and PRC2 suggests that their activities are also required for full differentiation (Leeb et al., 2010).

Decreased H3K27me3 and PcG binding has been observed at lineage-specific genes during neural differentiation (Bracken et al., 2006), keratinocyte maturation (Sen et al., 2008), and skeletal muscle differentiation (Caretti et al., 2004), and in neural progenitor cells (Mikkelsen et al., 2007). Several mechanisms have been proposed to account for these changes. For example, induction of the H3K27me3 histone demethylase Jmjd3 occurs during differentiation in the epidermis (Sen et al., 2008) and the expression of the PRC2 catalytic component Ezh2 is downregulated during skeletal muscle differentiation (Caretti et al., 2004) in a manner that is at least partially dependent on miR-214 expression (Juan et al., 2009). MicroRNAs (miRNAs) are a class of small noncoding RNAs that function as posttranscriptional regulators of gene expression (Brodersen and Voinnet, 2009; He and Hannon, 2004). miRNAs are involved in fine-tuning the expression of key target genes and play important roles in the regulation of ESC pluripotency and differentiation (Melton and Blelloch, 2010). However, to our knowledge, there have been no reports describing the regulation of PRC1 components by miRNAs during ESC differentiation.

In this study, we identify Cbx7 as the specific Pc “reader” of the H3K27me3 mark in ESCs. Cbx7 is expressed at high levels in ESCs and teratocarcinoma cells while its levels decrease during differentiation. This is in contrast to the expression pattern of other Pc proteins, such as Cbx2, Cbx4, and Cbx8, which are negatively regulated by Cbx7. Cbx7 has a critical role in maintaining ESC self-renewal and inhibiting differentiation and X-inactivation. We further identify miRNAs of the miR-125 and miR-181 families as key regulators of Cbx7 during ESC differentiation. Taken together, our results suggest distinct, context-dependent roles for individual PRC1 subunits and highlight the importance of Cbx7 and its regulatory miRNA network in ESC self-renewal and differentiation.

## Results

### The Pc Ortholog Cbx7 Is Associated with Pluripotency

**Figure 1:**
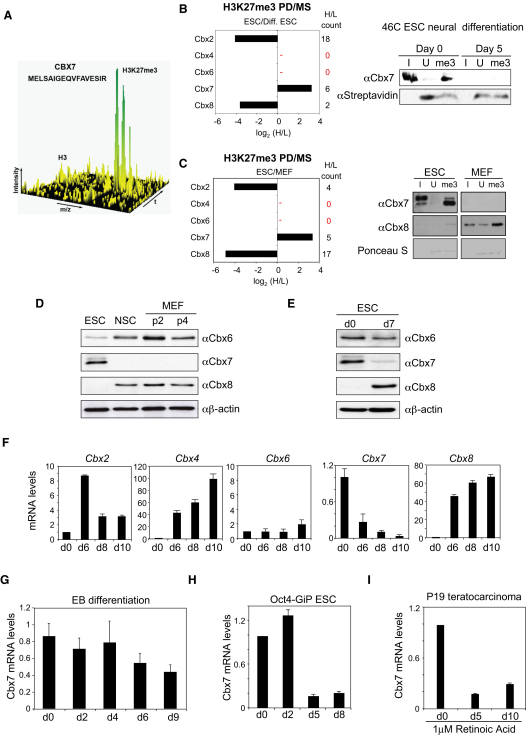
Cbx7 Expression Is Associated with Pluripotency (A) 3D visualization of Cbx7 binding to H3K27 versus H3K27me3 in a histone peptide PD experiment with SILAC-labeled mouse ESC nuclear extracts. The x axis represents the mass-to-charge ratio of the peptides (m/z), the chromatographic retention time (t) is plotted on the y axis, and intensity of the peptides is in the z axis. The identified Cbx7 peptide is ∼8 times more abundant in the “heavy” form compared to the “light” form, indicating specific H3K27me3 binding. (B and C) H3K27me3 is bound preferentially by Cbx7 in ESCs and by Cbx2 and Cbx8 in differentiated ESCs and MEFs. H3K27me3 histone peptide PDs were performed with SILAC-labeled ESC (heavy) and differentiated ESC (B, light) or MEF (C, light) nuclear extracts. A negative value corresponds to preferential binding in differentiated ESCs (B) or MEFs (C), and a positive value, to preferential binding on ESCs. The H/L spectral count is shown. Peptides corresponding to Cbx4 or Cbx6 were not detected. Peptide PD followed by western blot against Cbx7 and Cbx8 was performed to confirm the MS results. Streptavidin immunobloting or PonceuS staining revealing the biotinylated peptides that served as loading controls. I, input; U, unmethylated Histone H3 peptide; me3, H3K27me3 peptide. (D) Expression of Cbx6, Cbx7, and Cbx8 in different mouse cell types was assessed by immunoblotting. NSC, neural stem cells; MEF, mouse embryo fibroblasts at passage 2 (p2) or 4 (p4). (E) Cbx7 expression decreases upon neural differentiation of 46C ESCs. (F) qRT-PCR analyses of the expression of Pc orthologs in 46C cells at days 0, 6, 8, and 10 of neural differentiation. (G–I) The expression of Cbx7 decreases during embryoid body (EB) differentiation of 46C ESCs, neural differentiation of Oct4-GiP ESCs, and RA-induced differentiation of P19 mouse teratocarcinoma cells.

A defining feature of *Drosophila* Pc and its mammalian orthologs, Cbx2, Cbx4, Cbx6, Cbx7, and Cbx8, is the ability to bind to H3K27me3 via sequences in the conserved chromodomain. To investigate which of the Pc orthologs is responsible for PRC1 function in mouse ESCs, we took a quantitative proteomic approach (Vermeulen et al., 2010) to identify proteins that bind specifically to H3K27me3. One of the main binders, and the only Pc ortholog detected, was Cbx7 (Figure 1A). This was in sharp contrast to the H3K27me3 interactome of HeLa cells, which identified Cbx2, Cbx4, and Cbx8, but not Cbx7 (Vermeulen et al., 2010). To assess whether the binding of Cbx7 to H3K27me3 was specific for ESCs, we performed H3K27me3 pulldown (PD) assays using nuclear extracts from mouse ESCs grown in “heavy” medium and differentiated ESCs or mouse embryo fibroblasts (MEFs), grown in “light” medium. Mass spectroscopy analysis revealed that while Cbx7 was the only Pc ortholog recovered from ESCs, Cbx2 and Cbx8 were the primary binders of this modification detected in differentiated ESCs or MEFs (Figures 1B and 1C and Table S1 available online). These results were confirmed by peptide PD followed by immunoblot detection of Cbx7 or Cbx8 (Figures 1B and 1C and Figure S1A).

To explore the biology underlying the differences in Cbx affinity for H3K27me3, we analyzed the expression dynamics of Cbx7 and the other Pc proteins during the differentiation of ESCs (46C cells). These cells contain a Sox1-GFP transgene and upon neural differentiation, expression of the Sox1 reporter is induced concomitant with the downregulation of pluripotency genes that include Oct4 and Nanog (data not shown). Immunoblotting showed that while Cbx7 is expressed in the ESCs, very low levels were detected in the differentiated cells or in other cell types (Figures 1D and 1E). Expression of Cbx6 did not change significantly, whereas Cbx8 was absent in ESCs but induced upon differentiation (Figures 1D and 1E). Analysis of mRNA levels by qRT-PCR confirmed a sharp decline in Cbx7 expression during ESC differentiation as well as lower levels in NSCs (Figure 1F and Figure S1B). This was in stark contrast to the expression dynamics of other Pc paralogs, which were either unchanged (Cbx6) or upregulated (Cbx2, Cbx4, and Cbx8) during ESC differentiation (Figure 1F). We noted similar changes in Cbx7 expression when 46C ESCs were induced to differentiate into embryoid bodies (EBs) in an independent ESC line (Oct4-GIP ESC), and during retinoic acid-induced differentiation of P19 murine teratocarcinoma cells (Figures 1G–1I and Figures S1C–S1E). Taken together, these findings suggest that among the mammalian Pc homologs, Cbx7 is specifically associated with pluripotency.

### PRC1 Complex Composition Changes during ESC Differentiation

**Figure 2:**
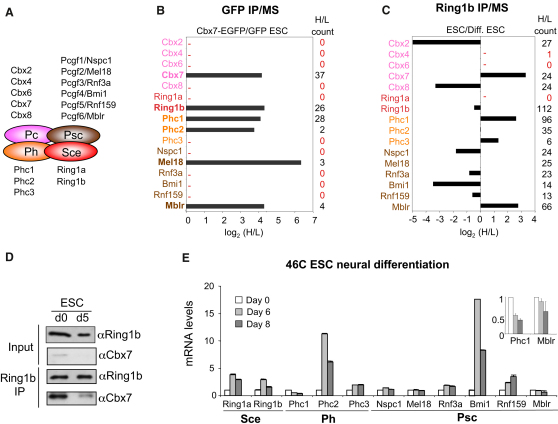
Changes in PRC1 Composition during ESC Differentiation (A) Putative PRC1 members in mouse cells: five Polycomb (Pc), six Posterior sex comb (Psc), three Polyhomeiotic (Ph), and two Sex combs extra (Sce) paralogs in mouse cells. (B) Cbx7-EGFP (heavy) and EGFP (light) ESCs were SILAC-labeled and subjected to GFP IP followed by MS analysis. The H/L peptide count is represented to the right. (C) ESCs (heavy) and differentiated ESCs (light) were SILAC-labeled and subjected to IP using αRing1b antibodies followed by MS analysis. Proteins preferentially binding to Ring1b in ESCs show a positive log_2_(H/L) value, and those that are preferentially associated to Ring1b in differentiated ESCs show a negative value. H/L spectral count is presented to the right. A cutoff of two detected peptides was used to reliably quantify protein ratios. (D) Cellular extracts were prepared from ESCs (d0) or ESCs subjected to neural differentiation (d5), and used for IP using αRing1b antibodies. Inputs and Ring1b IPs were subjected to immunoblots (IBs) to detect Ring1b and Cbx7. (E) The expression of PRC1 components during neural differentiation of 46C ESCs was analyzed by qRT-PCR. A different scale is used in the inset to better note how the expression of Phc1 and Mblr decreases upon ESC differentiation.

Cbx7 is one of five Pc orthologs that can be present in the core PRC1 complex (Figure 2A). Given the dynamic expression of Cbx7, we investigated the composition of Cbx7-containing PRC1 complexes in ESCs and during differentiation. To this end, we generated ESCs (derived from PGK12.1) expressing a Cbx7-EGFP protein (Figure S2A) and used a SILAC strategy to identify proteins that copurify with Cbx7-EGFP on GFP nanotrap beads. We detected two Psc orthologs (Mel18 and Mblr), two Ph proteins (Phc1 and Phc2), and one Sce protein (Ring1b), consistent with formation of canonical PRC1 complexes (Figure 2B and Table S2). As an alternative strategy, we used a monoclonal antibody against Ring1b to immunoprecipitate endogenous PRC1 complexes from ESCs prior to and following differentiation. Nuclear extracts from ESCs grown in heavy medium were compared to extracts from differentiated ESCs grown in light medium. Importantly, Cbx7 was the only Pc ortholog associated with Ring1b in undifferentiated ESCs. The differential association of Cbx7 with Ring1b was confirmed by immunoblot (Figure 2D). Consistent with the GFP IPs, Mblr and Phc1 were also found preferentially associated with Ring1b in ESCs. In contrast, Cbx2, Cbx8, Bmi1, and others were more prominently associated with Ring1b in differentiated cells. Finally, other PRC1 components such as Mel18 and Phc2 bound Ring1b to a similar extent in ESCs and differentiated cells. Both of these proteins were also detected in association with Cbx7 in ESCs (Figure 2C and Table S2).

To further understand the differential composition of PRC1 complexes, we analyzed the expression of PRC1 components during neural differentiation of 46C ESCs. Interestingly, we observed that the expression of Phc1 and Mblr, which were associated with Ring1b preferentially in ESCs, declined during ESC differentiation, thus mimicking the changes in Cbx7 expression (Figure 2E, inset). In contrast, the expression of other PRC1 members, most notably Bmi1 and Phc2, increased during neural differentiation (Figure 2E). These results are consistent with our observations in NSCs, an independent ESC line, and teratocarcinoma cells (Figures S2B–S2D).

### Dynamic Interplay between Cbx7 and Cbx8 during ESC Differentiation

**Figure 3:**
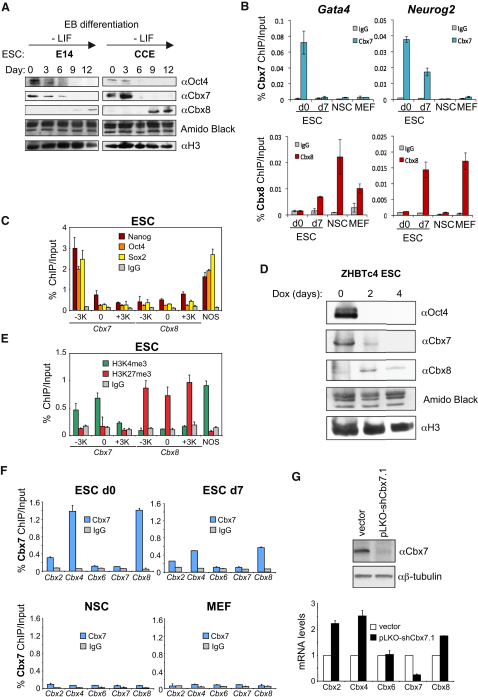
Dynamic Interplay between Cbx7 and Cbx8 Expression during Differentiation (A) Male (E14, CCE) ESCs underwent EB differentiation over 12 days and the levels of Oct4, Cbx7, Cbx8, and histone H3 in the chromatin fraction were assessed by IB. (B) ChIP analysis showing binding of Cbx7 and Cbx8 at the *Gata4* and *Neurog2* loci in ESCs, differentiated ESCs (d7), NSCs, and MEFs. Similar trends were observed with multiple primer sets for each locus (data not shown; primer sets listed in the Supplemental Information). (C) ChIP data showing Nanog, Oct4, and Sox2 binding upstream of *Cbx7*, but not *Cbx8*, in ESCs. NOS, positive control region 200 bp upstream of the *Nanog* gene bound by Nanog, Oct4, and Sox2. (D) IB for Oct4, Cbx7, Cbx8, and H3 following doxycycline (Dox)-induced repression of Oct4 in ZHBTc4 ESCs. Amido Black staining of the membrane is shown as a loading control. (E) ChIP data showing H3K4me3 and H3K27me3 histone modifications on the *Cbx7* and C*bx8* genes in ESCs. (F) ChIP analysis of Cbx7 binding at the promoters of Pc paralogs in ESCs. (G) Knockdown of Cbx7 in ESCs using shRNA results in upregulation of Cbx2, Cbx4, and Cbx8 mRNA expression.

The changes we observed above suggested a degree of antagonism in the expression of some PRC1 components during differentiation, particularly among the Pc orthologs. To substantiate this idea, we analyzed the levels of Cbx7 and Cbx8 in a panel of ESCs induced to differentiate into EBs (Figure 3A) or with retinoic acid (Figure S3A). Again, we observed that the Pc paralog expressed in ESCs is Cbx7, while Cbx8 is expressed upon differentiation. To further dissect the implications of this switch, we performed ChIP to determine the occupancy of Cbx7 and Cbx8 at four targets genes (Gata4, Sox3, Neurog2, and Nr2f2) in ESCs and their differentiated counterparts (Figure 3B and Figure S3B). Whereas Cbx7 was present at the promoter of each target gene in ESCs, it was barely detectable in differentiated ESCs, neural stem cells (NSCs), or MEFs. Cbx8 showed a reciprocal binding pattern, being absent from the promoters studied in ESCs but present in some of the differentiated cells in a gene-specific manner (Figure 3B and Figure S3B).

Analysis of published data sets (Marson et al., 2008) suggested that Cbx7 is unique among the Pc orthologs in being a target for the TFs of the pluripotency network, Sox2, Oct4, and Nanog (Figure S3C). ChIP studies using ESCs showed that indeed Sox2, Oct4, and Nanog all bind to a region upstream of the transcription start site (TSS) of Cbx7 (Figure 3C). Taking advantage of ZHBTc4 ESCs, which contain a tetracycline-regulated Oct4 transgene, we could show that depletion of Oct4 resulted in downregulation of Cbx7 expression (Figure 3D), consistent with Cbx7 being under control of the pluripotency TF network. We also observed a modest upregulation of Cbx8 in these cells. This is consistent with ChIP studies demonstrating that Cbx7 is marked by H3K4me3 in ESCs, while Cbx8 is marked by H3K27me3 (Figure 3E). An independent data set (Mikkelsen et al., 2007) also indicated that Cbx8 is a PcG target in ESCs (data not shown). Our ChIP assays confirmed that the promoter of Cbx8, and also those of Cbx2 and Cbx4, are occupied by Cbx7 in ESCs (Figure 3F), whereas neither Cbx7 nor Cbx6 register as a PcG target in the cell types examined. Finally, lentiviral shRNA-mediated knockdown of Cbx7 in ESCs resulted in increased expression of Cbx2, Cbx4, and Cbx8 (Figure 3G), suggesting that the expression of these Pc paralogs observed upon ESC differentiation may be directly caused by loss of Cbx7-mediated repression.

### Cbx7 Contributes to the Maintenance of Pluripotency in Mouse ESCs

**Figure 4:**
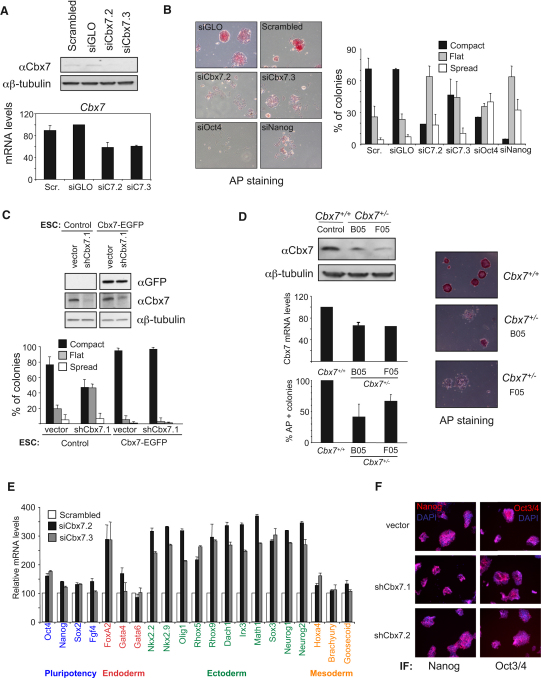
Cbx7 Levels Contribute to Maintenance of Pluripotency in ESCs (A) ESCs were transfected with siRNAs targeting Cbx7, and 3–5 days after transfection, Cbx7 expression was quantified by IB (upper panel) and qRT-PCR (lower panel). (B) Three days after transfection of ESCs with the indicated siRNAs, pluripotency was examined by AP staining. Representative images are shown. siGLO and a scrambled siRNA (AllStars) were used as a negative controls. Percentage of AP-positive colonies that showed a compact, flat, or spreading morphology is represented. (C) A lentiviral shRNA targeting Cbx7 3′UTR does not induce differentiation of Cbx7-EGFP ESCs. Control or Cbx7-EGFP ESCs were infected with an empty vector (vector) or a lentiviral shRNA targeting Cbx7 in its 3′UTR (shCbx7.1). Immunoblot showing the expression of the EGFP-Cbx7 fusion, endogenous Cbx7, or β-tubulin is shown (upper panel). Percentage of AP-positive colonies that showed a compact, flat, or spread morphology in the experiments are plotted. (D) Two independent *Cbx7^+/−^* ESC clones show spontaneous ESC differentiation. The expression of Cbx7 was assessed by IB (top panel) and qRT-PCR (middle). The number of AP-positive colonies was quantified (bottom). Representative pictures are shown (right). (E) Expression of a subset of pluripotency-associated and PcG target genes in ESCs transfected with Cbx7 siRNAs was monitored by qRT-PCR. (F) The expression of Nanog, Oct3, and Oct4 remains unchanged in ESCs infected with lentiviral vectors targeting Cbx7, as assessed by immunofluorescence.

To directly assess the role of Cbx7 in ESC pluripotency, we knocked down Cbx7 using two independent siRNAs (Figure S4A). The extent of the knockdown was validated by western blot and qRT-PCR (Figure 4A) and resulted in a clear and reproducible phenotype with a higher proportion of ESC colonies displaying a flattened or spread morphology indicative of a loss of ESC characteristics (Figure 4B). This phenotype was similar to the differentiation effects observed with siRNAs against Oct4 and Nanog (Figure 4B). In addition, the number of cells that were positive for alkaline phosphatase (AP), a marker of undifferentiated cells, was reduced upon Cbx7 knockdown (data not shown). Similar results were observed with two additional shRNAs delivered by lentiviral vectors (Figures S4B and S4C). To ensure that off-target effects were not responsible for these phenotypes, we used an shRNA (pLKO-shCbx7.1) that targets the 3′UTR of Cbx7 and repeated the experiment in ESCs expressing the Cbx7-EGFP protein (expression of this construct lacking the 3′UTR was unaffected by this shRNA) (Figure 4C). Consistent with a Cbx7-mediated phenotype, infection with the pLKO-shCbx7.1 lentivirus resulted in differentiation of control ESCs but not of Cbx7-EGFP-expressing ESCs (Figure 4C). To confirm by an alternative method that Cbx7 contributes to ESC pluripotency, we obtained two independent clones of ESCs in which one allele of Cbx7 has been inactivated (Wellcome Trust Sanger Institute's knockout mouse project, http://www.komp.org). As expected, these *Cbx7^+/−^* ESC clones (B05 and F05) expressed lower levels of Cbx7 as assessed by qRT-PCR and immunoblot (Figure 4D). We observed a lower proportion of AP-positive cells among *Cbx7^+/−^* ESCs than in control *Cbx7^+/+^* ESCs (Figure 4D). Interestingly, delivery of a FLAG-tagged version of Cbx7 into *Cbx7^+/−^* ESC resulted in an increased number of colonies with a compact ESC morphology (Figures S4D and S4E).

Finally, in order to understand how the depletion of Cbx7 affected ESCs, we analyzed the expression of several Pc target genes associated with differentiation, and observed that their expression increased upon Cbx7 knockdown, particularly that of ectoderm-lineage-associated genes (Figure 4E), whereas several pluripotency-related genes remained relatively unaffected at both the mRNA (Figure 4E) and protein (Figure 4F and Figure S4F) level. This suggests that although depletion of Cbx7 results in a degree of spontaneous differentiation, ESCs that have low levels of Cbx7 can still self-renew.

### Cbx7 Expression Promotes ESC Self-Renewal by Suppressing Differentiation and X-Inactivation

**Figure 5:**
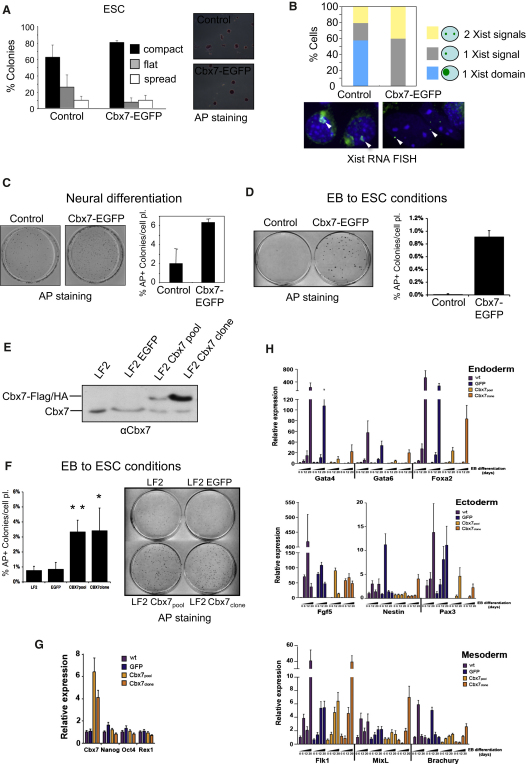
Cbx7 Expression Blocks ESC Differentiation and X-Inactivation (A) PGK12.1 ESCs expressing Cbx7-EGFP and control ESCs were kept in ESC media under nondifferentiation conditions. Cells were stained with AP. Representative images and the percentage of AP-positive colonies showing a compact, flat, or spread morphology are shown. (B) Control and Cbx7-EGFP-expressing ESCs were subjected to 5 days of retinoic acid treatment to induce differentiation. Xist RNA was detected by RNA FISH, and more than 100 cells were counted for each clone. Represented is the percentage of cells showing two (yellow) or one (gray) punctate Xist RNA signals, or a properly inactivated Xist RNA domain (blue). (C) Control and Cbx7-EGFP ESCs were subjected to neural differentiation for 4 days and stained with AP. Representative images were taken and the percentage of AP-positive colonies of the total cells plated is represented. (D) Control and Cbx7-EGFP-expressing ESCs were cultured in nonattachment conditions without leukemia inhibitory factor to form EBs. EBs were then dissociated and plated back in ESC medium at day 20. Numbers of ESC-like colonies were analyzed by AP staining after 5 days. (E) LF2 ESCs, expressing EGFP (control), and Cbx7-FLAG/HA (pool and clone) were probed for levels of endogenous and exogenous Cbx7 protein. (F) LF2 cells described above were assayed as in (D). Percentage of AP-positive colonies formed per cells plated is represented. ^∗^p < 0.005, ^∗∗^p < 0.00005. (G and H) Cbx7 expression in ESCs prevents induction of lineage markers upon differentiation. (G) mRNA expression of Cbx7 and pluripotency factors in LF2 ESCs (wt) and LF2 ESCs expressing EGFP (control) and Cbx7-FLAG/HA (pool and clone) by qRT-PCR. (H) mRNA expression of endoderm, mesoderm, and ectoderm markers in EBs derived from above cells analyzed by qRT-PCR at days 6, 12, and 20.

The fact that Cbx7 levels decreased during ESC differentiation prompted us to test whether ectopic expression of Cbx7 could sustain pluripotency. When cultured under ESC conditions, ESCs overexpressing Cbx7 displayed lower levels of spontaneous differentiation, as judged by the number of colonies with a compact morphology (Figure 5A) or stained positively for Nanog (not shown). When cells were placed under differentiation conditions, we noticed striking effects resulting from ectopic Cbx7 expression. First, Cbx7 expression resulted in the dramatic inhibition of X chromosome inactivation during ESC differentiation, as measured by quantification of nuclear Xist RNA expression in several independent female ESC clones (Figure 5B and Figure S5A). The cells also retained ESC characteristics when subjected to neural differentiation or EB formation, as evaluated by AP, Oct4, and Nanog staining as well as qRT-PCR analysis (Figures 5C and 5D and Figures S5B–S5D).

To exclude the possibility that these results were clone specific or due to excessive levels of Cbx7, we expressed a FLAG/HA-tagged version of Cbx7 in ESCs and derived both a pool, which expressed lower overall levels of Cbx7, and a clone expressing higher levels of Cbx7 (Figure 5E). These ESCs, which were subjected to EB differentiation and replating into ESC conditions, also gave rise to significantly more AP-positive colonies than did the corresponding controls (Figure 5F and Figure S5E). We also analyzed the expression of several pluripotency and lineage-specific genes at different times during differentiation (Figures 5G and 5H). While Cbx7 did not affect the basal expression of pluripotency-associated genes in the ESCs (Figure 5G), it prevented or delayed the induction of most of the differentiation-associated genes representative of endoderm, mesoderm, and ectoderm (Figure 5H). Collectively, ectopic expression of Cbx7 results in increased ESC self-renewal and prevents ESC differentiation and X chromosome inactivation.

### miRNA Families miR-125 and miR-181 Are Bona Fide Cbx7 Regulators

In identifying a key role for Cbx7 in pluripotency, it was important to consider the mechanisms responsible for the downregulation of Cbx7 during differentiation. Although this could obviously reflect transcriptional control, we speculated that Cbx7 could additionally be subjected to posttranscriptional regulation by miRNAs.

**Figure 6:**
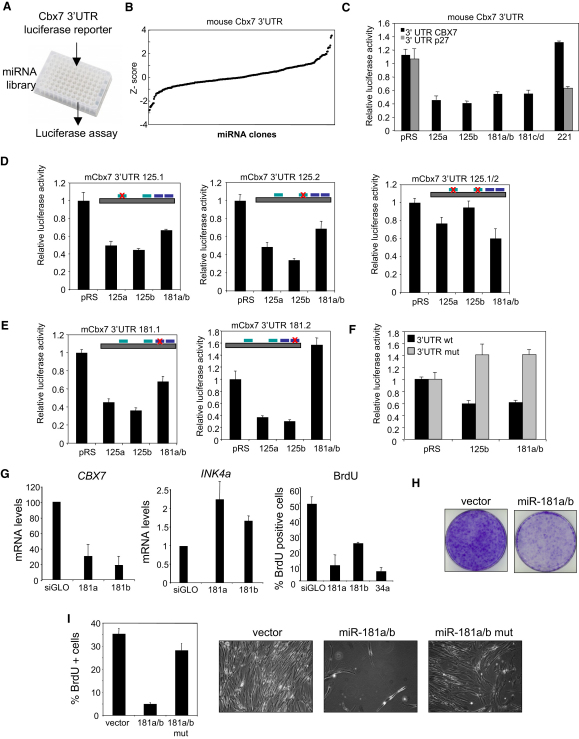
The miR-125 and miR-181 miRNA Families Are Bona Fide Cbx7 Regulators (A) A mouse Cbx7-3′UTR luciferase reporter was cotransfected in HEK293T cells with a miRNA library in 96-well format to screen for Cbx7 regulatory miRNAs. (B) Results of the miRNA screen were plotted and Z-scores were calculated. miRNAs with Z-scores lower than −2 were chosen for retesting. (C) Validation of miRNAs identified in the screen confirms the miR-125 and miR-181 families as regulators of mouse Cbx7 3′UTR in a luciferase reporter assay. miR-221 regulation of a p27-3′UTR reporter is included as a control. (D) Luciferase assays using reporters in which the two putative miR-125 target sites of the Cbx7 3′UTR have been mutated individually or combined. (E) Luciferase assays using reporters in which two putative miR-181 target sites in the Cbx7 3′UTR have been mutated. (F) A Cbx7-3′UTR reporter with all putative miR-125 and miR-181 sites mutated (3′UTR mut) is resistant to regulation by the miR125 and miR181 families. (G) IMR90 cells were transfected with miR-181a, miR-181b, or miR-34 mimics, and the expression of *CBX7* (left) and *INK4a* mRNA (middle) was monitored by qRT-PCR. The percentage of BrdU-positive cells in the same experiment was monitored by immunofluorescence and quantified (right). (H) Expression of miR-181a/b in human IMR90 fibroblasts results in decreased cell growth as assessed by crystal violet staining. (I) IMR90 cells were infected with miR-181a/b or a version in which the seed sequences had been mutated. Their ability to induce senescence was assessed by BrdU incorporation (left) and phase microscopy (right).

To identify miRNAs regulating Cbx7 expression, we used a mouse Cbx7-3′UTR reporter (psiCHECK2-Cbx7-3′UTR) and a miRNA expression library comprising 371 miRNAs (Voorhoeve et al., 2006) to perform a reporter screen in single-well format (Figure 6A). We conducted two independent screens and Figure 6B shows the results obtained in one of the replicas. We set the Z-score threshold at < −2 to select miRNAs significantly downregulating the Cbx7 reporter. The candidates were miRNAs of the miR-125 (miR-125a and miR-125b) and the miR-181 (a vector expressing miR-181a and miR-181b and another expressing miR-181c and miR-181d) families. miRNAs from these families have two predicted binding sites in the 3′UTR of Cbx7 (see scheme in Figure S6A) that are conserved among vertebrates (data not shown). To verify the results from the screening, we retested vectors expressing miR-125a, miR-125b, miR-181a/b, and miR-181c/d and confirmed their ability to reduce the luciferase activity when cotransfected with psiCHECK2-Cbx7-3′UTR (Figure 6C). Members of the miR-125 and miR-181 families also regulated human CBX7 in similar reporter assays (Figure S6B).

To determine which of the putative miRNA target sites were responsible for the regulation of Cbx7, we generated reporter constructs bearing specific mutations (Figure S6A). While mutation of both miR-125 target sites was required to preclude downregulation of the Cbx7-3′UTR reporter by miR-125 (Figure 6D), only the second miR-181 target site was important for downregulating Cbx7 (Figure 6E). We also generated a Cbx7-3′UTR reporter in which the four putative miR-125 and miR-181 target sites were mutated (Cbx7-3′UTR mut). This mutant reporter was resistant to downregulation by miRNAs of both the miR-125 and miR-181 families (Figure 6F). Similarly, deletion of the seed sequences of miRNA-181a/b (miR-181a/b mut) or miRNA-125b (miR-125b mut) abrogated the ability of these constructs to downregulate the Cbx7-3′UTR reporter (Figure S6C).

### Expression of miRNA-181a/b Induces Senescence by Targeting Cbx7

One of the best-defined functions of Cbx7 is its ability to delay senescence through PRC1-mediated repression of *INK4a*. In human fibroblasts, shRNA-mediated knockdown of endogenous CBX7 impairs cell growth and induces premature senescence (Gil et al., 2004). To investigate whether the miR-125 and miR-181 families can act as bona fide regulators of Cbx7, we assessed their ability to cause p16^INK4a^-dependent senescence in human fibroblasts, either by transfecting the cells with miRNA mimics (Figure 6G) or by retroviral infection (Figure 6H and Figures S6D–S6G). Expression of miR-181a or miR-181b caused downregulation of Cbx7 (Figure 6G) accompanied by induction of p16^INK4a^ expression (Figure 6G and Figure S6F). Specificity was confirmed by using variants of miR-181a and miR-181b with a mutated seed sequence. These variants had no effect on Cbx7 levels or senescence induction (Figure 6I and Figure S6D). In similar experiments, miR-125b also caused a seed-sequence-dependent arrest correlated with upregulation of p16^INK4a^ (Figures S6E–S6G). Collectively, these results suggest that the two miRNA families identified in our screen are bona fide regulators of Cbx7 expression.

### miR-125 and miR-181 Regulate ESC Differentiation via Cbx7

**Figure 7:**
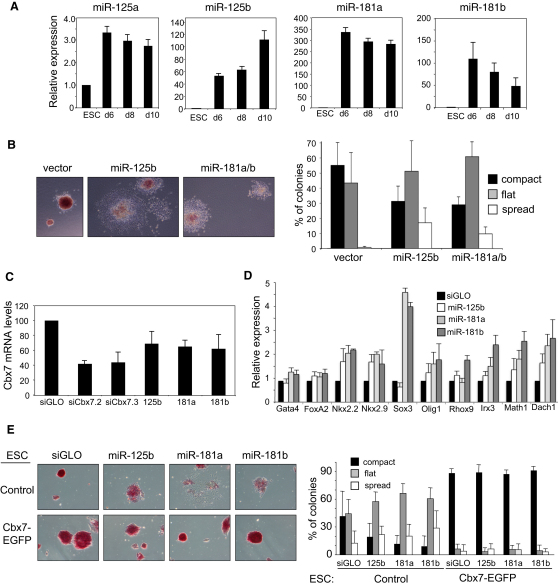
The miR-181 and miR-125 Families Regulate Cbx7 Expression and Influence ESC Differentiation (A) The expression of miR-125 and miR-181 families is upregulated during neural differentiation of 46C ESC as measured using Taqman probes. (B) Expression of miR-125b or miR-181a/b by retroviral infection causes a loss of ESC properties. Representative images of AP-stained cells (left) and quantification of colonies (right) are presented. (C) Transfection of ESCs with miRNA mimics for miR-125b, miR-181a, or miR-181b results in Cbx7 downregulation. (D) Expression of a subset of PcG target genes associated with differentiation in ESCs transfected with miR-125b, miR-181a, or miR-181b mimics were monitored by qRT-PCR. (E) Control ESCs (PGK12.1) or Cbx7-EGFP ESCs (PGK12.1 Cbx7-EGFP) were transfected with siGLO or synthetic mimics for miR-125b, miR-181a, and miR-181b. Cells were maintained in ESC media and stained with AP. Representative images (left) and the percentage of AP-positive colonies showing a compact, flat, or spreading morphology are shown (right).

Given these findings in fibroblasts, we next investigated whether members of the miR-125 and miR-181 families have a role in ESCs. Interestingly, the expression of most members of the miR-125 and miR-181 families is low or undetectable in 46C ESCs, but becomes upregulated upon neural differentiation (Figure 7A). The increased expression of these miRNAs upon differentiation was also observed in other ESC lines, during EB formation and upon differentiation of P19 teratocarcinoma cells (data not shown). The inverse correlation between the expression of the miR-125 and miR-181 families and Cbx7 suggested that these miRNAs could contribute to the regulation of Cbx7 levels during ESC differentiation.

Consistent with this hypothesis, expression of miR-125b or miR-181a/b in ESCs promoted differentiation as assessed by increased colonies with a flat or spread morphology (Figure 7B) and a reduced frequency of AP-positive colonies (Figure S7A). To confirm and extend these findings, we transfected mimics for miR-125b, miR-181a, or miR-181b into ESCs. The levels of the transfected miRNAs were similar to the levels of these miRNAs during ESC differentiation (Figure S7B) and downregulated Cbx7 expression (Figure 7C). Upon transfection with mimics for miR-125b, miR-181a, or miR-181b, we observed a loss of ESC characteristics (Figure 7E, see transfection in control ESCs, and Figure S7C). Furthermore, ESCs transfected with miRNA mimics for miR-125b, miR-181a, and miR-181b displayed increased expression of a subset of Pc target genes involved in lineage specification (Figure 7D).

To understand whether Cbx7 is a critical target of the miR-125 and miR-181 families during ESC differentiation, we compared the effect of transfecting miR-125b, miR-181a, or miR-181b mimics in control ESCs or those expressing Cbx7-EGFP lacking its 3′UTR, which are therefore resistant to miRNA regulation. In contrast to their effects in control ESCs, these miRNAs did not enhance the differentiation of Cbx7-EGFP ESCs (Figure 7E), even when the cells were switched to neural differentiation conditions (Figures S7D and S7E). Similar results were obtained upon infection of Cbx7-EGFP ESCs with retroviral vectors expressing miR-125b or miR-181a/b (Figure S7D). These results demonstrate that miR-125b and miR-181a/b are induced during ESC differentiation and contribute to this process by downregulating Cbx7 expression through direct targeting of its 3′UTR.

## Discussion

The rationale for the evolutionary expansion of PcG genes, particularly those encoding PRC1 components, is not well understood (Whitcomb et al., 2007). Here, we hypothesized that the numerous potential combinations of PRC1 in mammals might have specialized or context-dependent roles. In the few cases where PRC1 composition has been examined, such as at the *INK4a/ARF* locus in human fibroblasts, several variants of PRC1 have been found to colocalize, yet each component appears to contribute to the regulation of *INK4a* (Maertens et al., 2009). However, the variable phenotypes associated with genetic ablation of PRC1 components in mice imply that they are not functionally equivalent (Sparmann and van Lohuizen, 2006). In this study, we investigated which of the five mammalian orthologs of Pc is required for PRC1 function and maintenance of pluripotency in mouse ESCs.

In contrast to somatic cells or differentiated ESCs, Cbx7 is the Pc ortholog that reads H3K27me3 in ESCs. Although this could be explained by the high affinity of Cbx7 for H3K27me3 (Bernstein et al., 2006b; Yap et al., 2010), this is likely not the case, as we identified Cbx2 and Cbx8 as the Pc orthologs interacting with H3K27me3 in MEFs and differentiated ESCs. In addition, analysis of the expression of Cbx7 and the other Pc orthologs during ESC differentiation showed very different dynamics. While Cbx7 is expressed in ESCs and is sharply downregulated during differentiation, the expression of the other Pc orthologs did not change significantly (Cbx6) or in contrast to Cbx7, increased upon differentiation (Cbx2, Cbx4, and Cbx8). Interestingly, among the different Pc orthologs, the expression of Cbx7 was clearly associated with pluripotency; we observed that it is highly expressed in ESCs and teratocarcinomas. The expression of other PRC1 members, most notably Phc1 and Mblr, seem to also be associated with pluripotency, while others like Bmi1 and Phc2 were more highly expressed in differentiated ESCs or other cell types. As a result, subunit composition of PRC1 complexes changes in a dynamic fashion during ESC differentiation, as highlighted in our mass spectrometry (MS) experiments. Given the multiple possible combinations of PRC1 subunits and the potential difficulty in detecting some members due to experimental bias of the technique used and/or a lack of antibodies, we believe a combination of MS, gel filtration, immunoblotting, and other approaches should be used to thoroughly investigate the changes in composition and coexistence of different PRC1 complexes during ESC differentiation in the future.

Direct evidence for Cbx7 being a critical factor in pluripotency came from the finding that knockdown of Cbx7 accelerated ESC differentiation and correlated with increased expression of lineage-specific PcG targets. Conversely, ectopic expression of Cbx7 impaired ESC differentiation and X chromosome inactivation. This blockade suggested a prominent role for Cbx7 overexpression in repressing PRC1 target genes during ESC differentiation. A possible explanation for the predominance of Cbx7 in stem cells is that its expression is under the control of the pluripotency network of TFs. Consistent with this hypothesis, depletion of Oct4 in ESCs caused downregulation of Cbx7, and ChIP studies demonstrated direct occupancy by Oct4, Sox2, and Nanog. Interestingly Cbx2, Cbx4, and Cbx8, which show a reciprocal pattern of expression to that of Cbx7, are direct targets of Cbx7. The importance of Cbx7 in pluripotency is therefore underscored by its role in restraining other Pc orthologs in ESCs. Whether other PRC1 components are subjected to similar regulation remains to be investigated.

We suspected that posttranscriptional mechanisms might contribute to the downregulation of Cbx7 during ESC differentiation. A number of miRNAs have been implicated in regulating the balance between self-renewal and differentiation of ESCs, including those of the miR-302 and let-7 families (Melton and Blelloch, 2010). Moreover, Ezh2, the enzymatic component of the PRC2 complex, is regulated by miR-214 during differentiation of ESCs and muscle cells (Juan et al., 2009). Here we identified members of the miR-125 and miR-181 families as bona fide regulators of Cbx7. The expression of either miR-125b or miR-181a/b in human fibroblasts resulted in upregulation of p16^INK4a^ and senescence in agreement with a recent report showing that miR-125b can cause senescence in human melanoma cells (Glud et al., 2011). Importantly, miR-125b, miR-181a, and miR-181b are not expressed (or are expressed at low levels) in ESCs and are sharply induced during ESC differentiation. Previous reports have shown that miR-125 is induced in NSCs and targets lin28 to allow processing of let-7 (Rybak et al., 2008) and that regulation of multiple miR-125b targets is required for neural differentiation (Le et al., 2009). Similarly, miR-181 is upregulated during differentiation to myoblast and hematopoietic lineages (Chen et al., 2004; Naguibneva et al., 2006) and plays an active role in driving both processes. miR-181 targeting of HoxA11, which itself represses MyoD, is a key facet of myoblast differentiation (Naguibneva et al., 2006). Whether control of PRC1 function through targeting of Cbx7 could also play a role in these processes is presently unknown.

In conclusion, we have identified a prominent role for Cbx7 in pluripotency. Cbx7 function in ESCs is critical to suppress differentiation. A complex mechanism is clearly in place in order to fine-tune the expression of Cbx7 and its orthologs both in ESCs and upon differentiation. This involves transcriptional control of Cbx7 by the pluripotency network of TFs in ESCs as well as induction of miRNAs that target Cbx7 during differentiation. Future studies will explore how expression of the miR-125 and miR-181 families is controlled in ESC differentiation, and whether other PRC1 members are also regulated in a similar fashion.

## Experimental Procedures

### Plasmids

The miR-Vec library and control vector have been described by (Voorhoeve et al., 2006). The generation of the miRNA mutants, reporter plasmids, and Cbx7-derived plasmids used in this study is described in the Supplemental Information.

### Cell Culture and Differentiation Assays

P19, HEK293T, and IMR90 cells were maintained in Dulbecco's modified Eagle's medium (Invitrogen) with 10% fetal bovine serum (PAA) and 1% antibiotic-antimycotic solution (Invitrogen). Mouse ESC lines CCE, E14, ZHBTc4, LF2, PGK12.1, 46C, and Oct4-GIP, and *Cbx7^+/−^* lines (B05 and F05), were cultured as described in detail in the Supplemental Information. *Cbx7^+/−^* ESCs (B05 and F05) were obtained from the Wellcome Trust Sanger Institute.

### Retroviral and Lentiviral Infection

Virus production and infection have been described elsewhere (Banito et al., 2009).

### Mass Spectroscopy

This was performed essentially as described in Vermeulen et al. (2010). Details are mentioned in the Supplemental Information.

### Peptide PD and GFP and Ring1b Immunoprecipitation

These assays were conducted using standard protocols that are described in detail in the Supplemental Information.

### Colony Formation Assay after EB Disaggregation

ESCs were differentiated to form EBs under nonadherent conditions. At days 12 and 20, EBs were washed with PBS, trypsinized, and resuspended in ESC media. Cells were plated at 5,000, 10,000 or 15,000 cells per gelatinized plate. After 5–10 days, colonies were fixed and stained for AP. The number of colonies were counted from scanned images using Image J software, and plotted as percentage of AP-positive colonies per cells plated.

### AP Staining

ESCs were plated (5 × 10^4^) in 6-well plates and fixed with 4% paraformaldehyde for 1–2 min. Staining was performed using the Alkaline Phosphatase Detection Kit (Millipore or Stemgent) according to manufacturers' protocol.

### Reverse Transfection and Luciferase Assay

For the luciferase screening, HEK293T cells were reverse transfected using Polyethylenimine (PEI, Sigma) to individually transfect 371 clones from the miR-Vec library in a 96-well plate format. A 9:1 ratio of miR-Vec to luciferase reporter construct was used. miR-Vec-Ctrl was used as control vector. A 3:1 ratio of PEI to DNA was used, and after incubation of reagent-DNA complexes for 30 min, cells were added. *Firefly* and *Renilla* luciferase activities were measured using the Dual-Luciferase Reporter Assay system (Promega) 48 hr after transfection. Values were expressed as the number of median-adjusted standard deviations (Z-score value) and a threshold was established (Z-score < −2) to identify miRNAs that caused a significant reduction of luciferase expression when cotransfected with psiCHECK2-Cbx7-3′UTR. miRNAs with a Z-score value lower than −1 when cotransfected with empty psiCHECK were discarded.

### BrdU Assay and Crystal Violet Staining

BrdU labeling was performed for 16 hr. Crystal violet staining was performed as previously described (Banito et al., 2009).

### Immunofluorescence and Immunoblotting

Immunofluorescence was performed using an InCell Analyzer 1000 (GE). Image processing and quantification was performed using InCell Investigator software (GE). Immunoblotting was performed following standard procedures, and, when indicated, from chromatin fractions prepared as described (Bernstein et al., 2006b). Donkey anti-rabbit HRP (GE Healthcare) and sheep anti-mouse HRP (GE Healthcare) -conjugated antibodies were used and signals were detected by ECL (GE Healthcare). Antibodies are listed in the Supplemental Information.

### qRT-PCR Analysis

qRT-PCR was performed as described previously (Banito et al., 2009). A list of primers and Taqman probes used is presented as part of the Supplemental Information.

### RNA Interference and miRNA Transfection

IMR90 or 46C ESCs were transfected with 30 nM siRNA for IMR90 and 100 nM for 46C ESCs in 6-well plates. A 3.5% solution of HiPerFect transfection reagent (QIAGEN) was prepared in serum-free DMEM and then mixed with the siRNA. The mix was incubated for 30 min at room temperature and then added to the cells. Medium was changed on the following day and cells were either fixed for immunofluorescence or harvested for RNA extraction 24–96 hr later. The Cy3-labeled si*GLO* cyclophilin B siRNA (Dharmacon) was used to monitor transfection efficiency and as a negative control. A scrambled siRNA (AllStars) or *Silencer* Select Negative Control #1 and #2 siRNA (Ambion) were included as additional negative controls in most experiments. For a list of siRNAs used see the Supplemental Information.

### ChIP

ChIP experiments were performed as previously described (Maertens et al., 2009). A detailed explanation of the protocol and a list of the primers used for the ChIP is provided in the Supplemental Information.

### Xist RNA FISH

RNA FISH was performed as described in (Masui et al., 2011) and under “protocols”at the following URL: http://www.epigenesys.eu/.

## Supplemental Information

Document S1. Figures S1–S7 and Supplemental Experimental Procedures

Table S1. MS Data of the H3K27me3 Pulldown Experiments Shown in Figures 1B and 1C

Table S2. MS Data of the Ring1b and GFP Pulldown Experiments Shown in Figures 2B and 2C
