# Supplementary material for: MicroRNA Regulation of Cbx7 Mediates a Switch of Polycomb Orthologs during ESC Differentiation
Source: Cell Stem Cell. 2012 Jan 6;10(1-5):33–46. doi: 10.1016/j.stem.2011.12.004 (PMC3277884; doi:10.1016/j.stem.2011.12.004)
Supplement: Document S1. Figures S1–S7 and Supplemental Experimental Procedures [file mmc1.pdf]

Cell Stem Cell, volume *10*

Supplemental Information

MicroRNA Regulation of Cbx7 Mediates a Switch of Polycomb Orthologs during ESC Differentiation

Ana O'Loghlen, Ana M. Muñoz-Cabello, Alexandre Gaspar-Maia, Hsan-Au Wu, Ana Banito, Natalia Kunowska, Tomas Racek, Helen N. Pemberton, Patrizia Beolchi, Fabrice Lavial, Osamu Masui, Michiel Vermeulen, Thomas Carroll, Johannes Graumann, Edith Heard, Niall Dillon, Veronique Azuara, Ambrosius P. Snijders, Gordon Peters, Emily Bernstein, and Jesus Gil

SUPPLEMENTAL FIGURES

O'Loghlen *et al.* Sup Figure S1

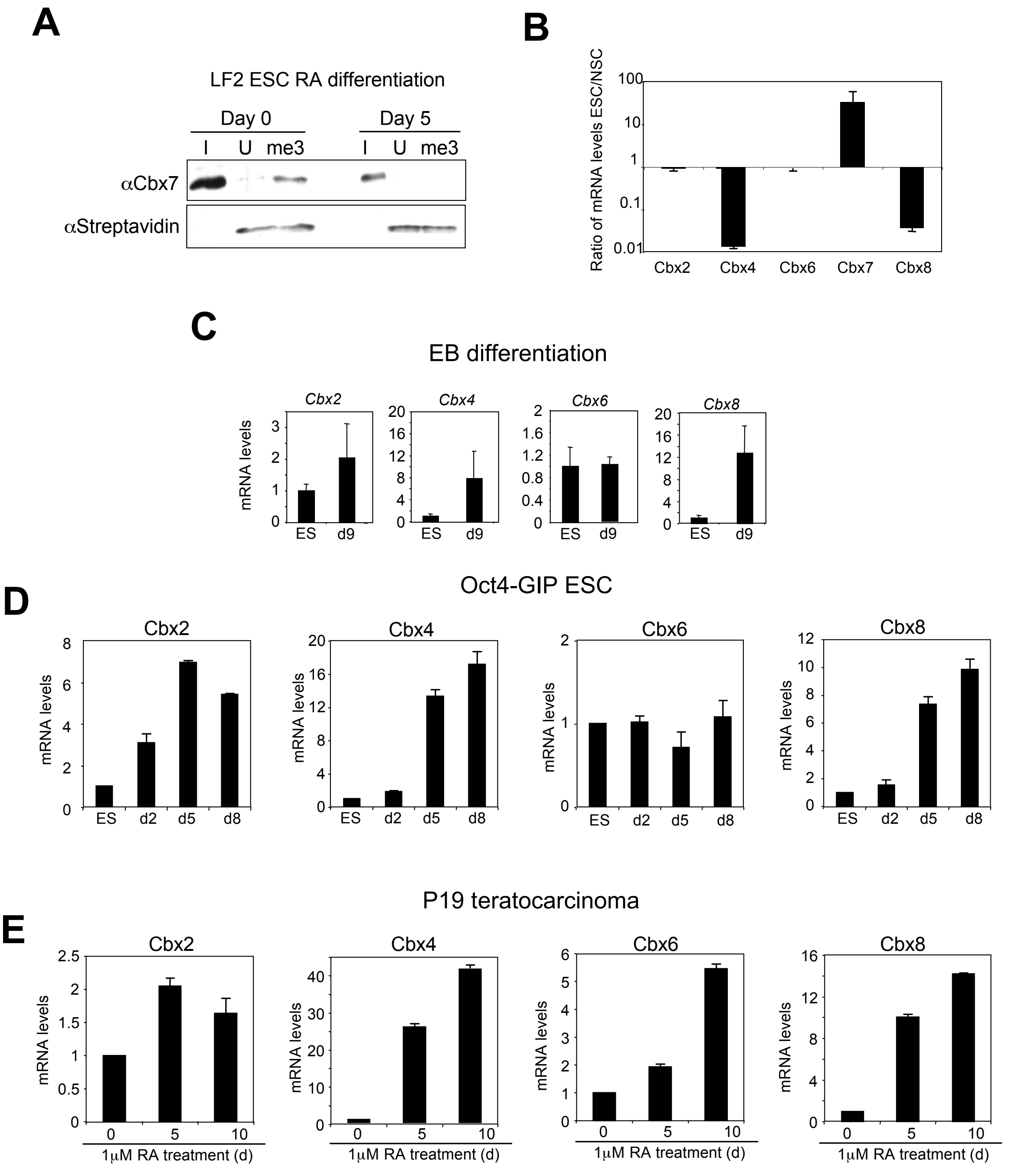

O'Loghlen *et al.* Sup Figure S2

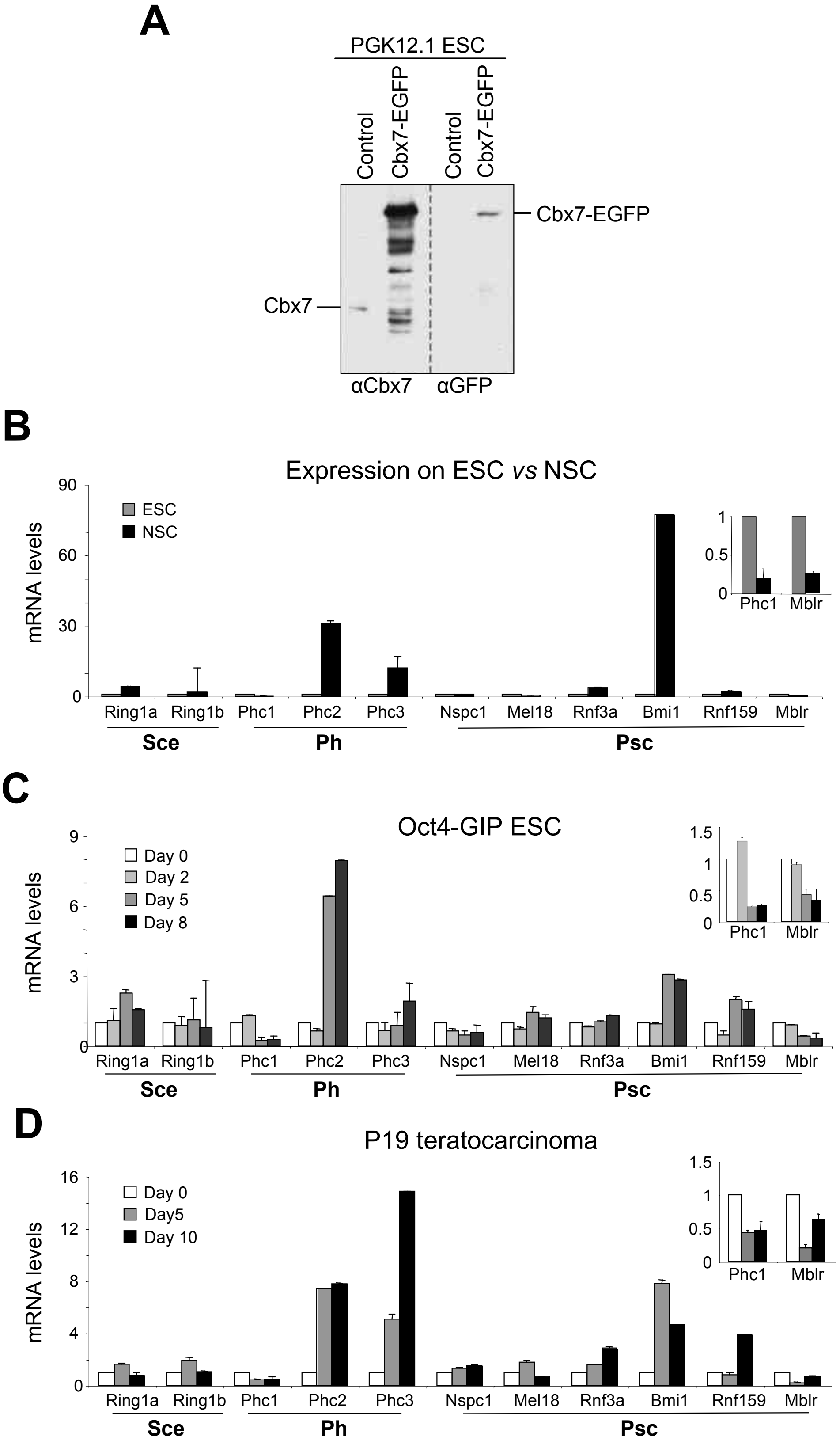

O'Loghlen *et al.* Sup Figure S3

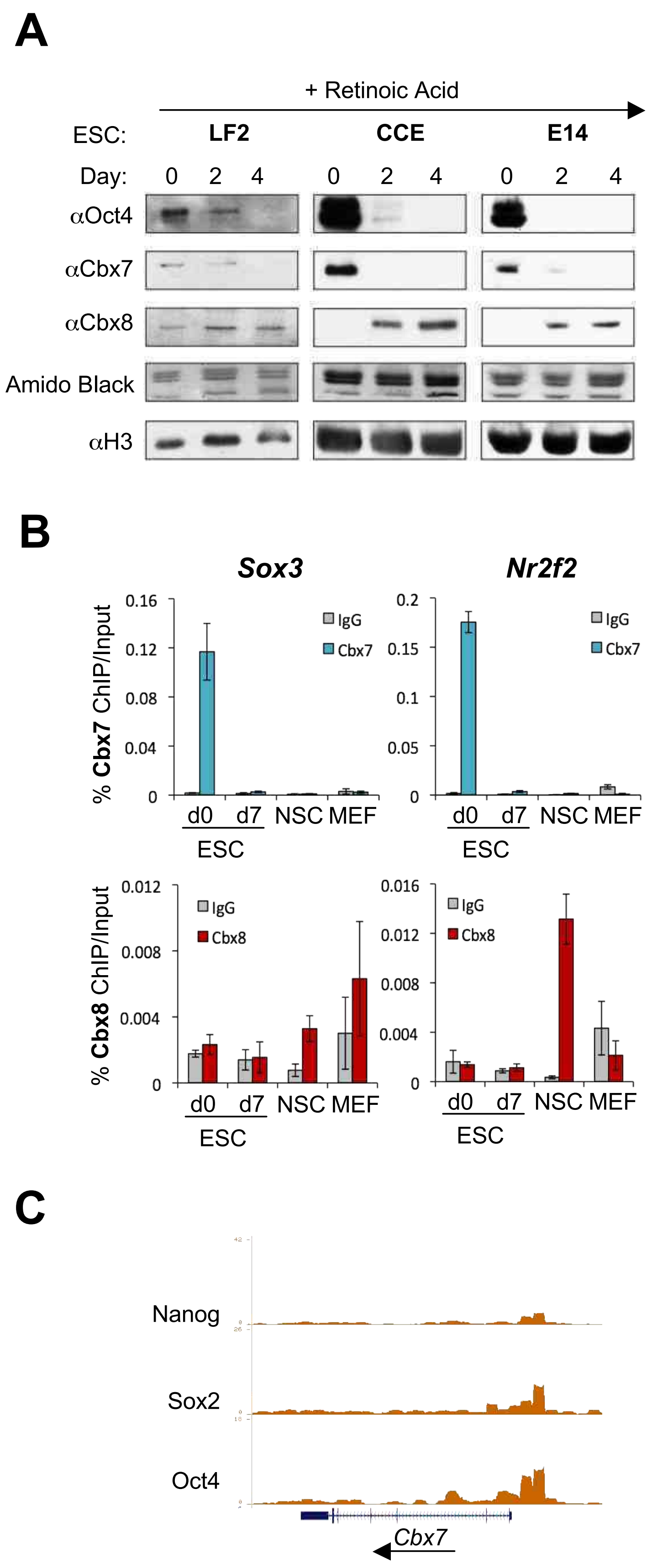

O'Loghlen *et al.* Sup Figure S4

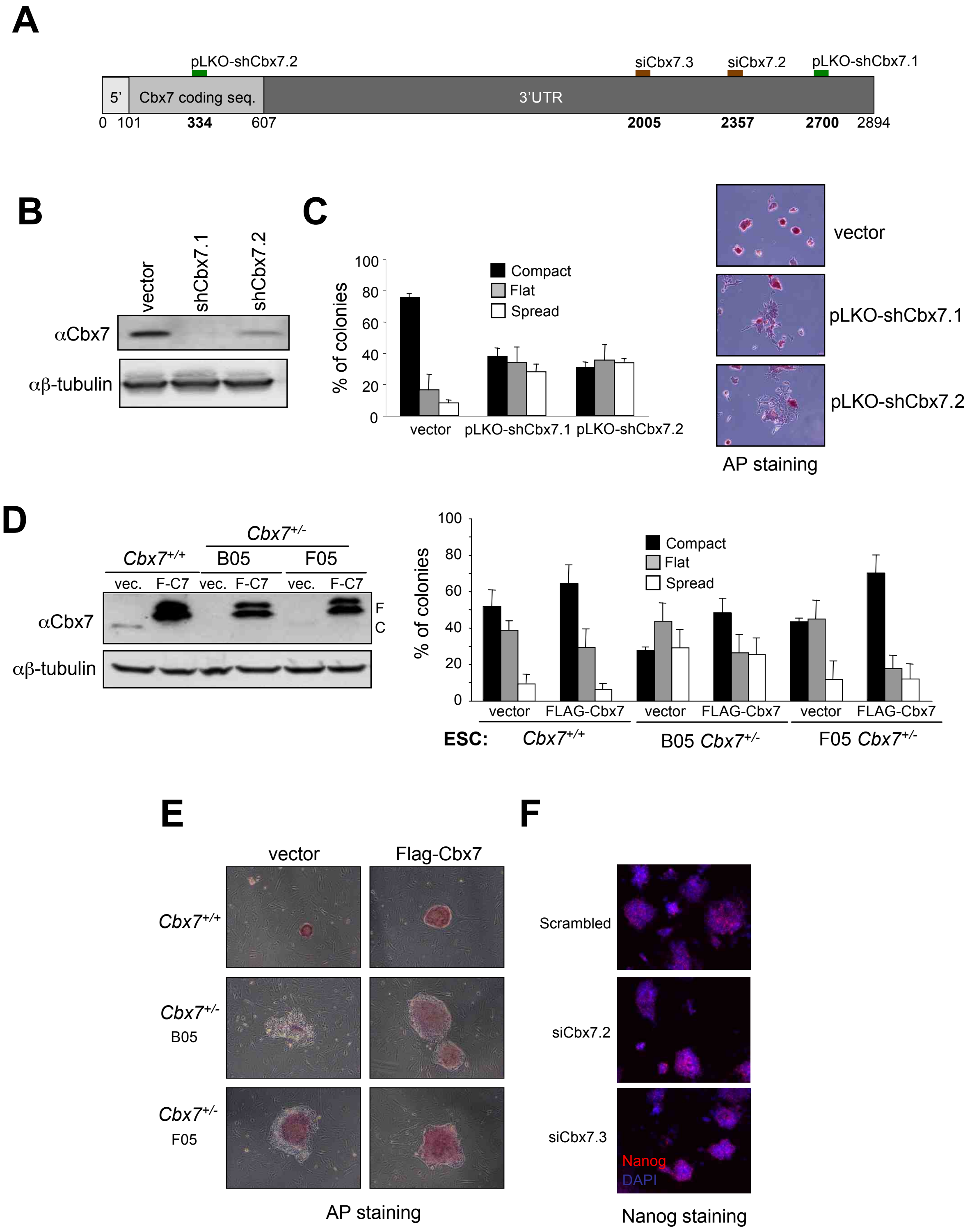

O'Loghlen *et al.* Sup Figure S5

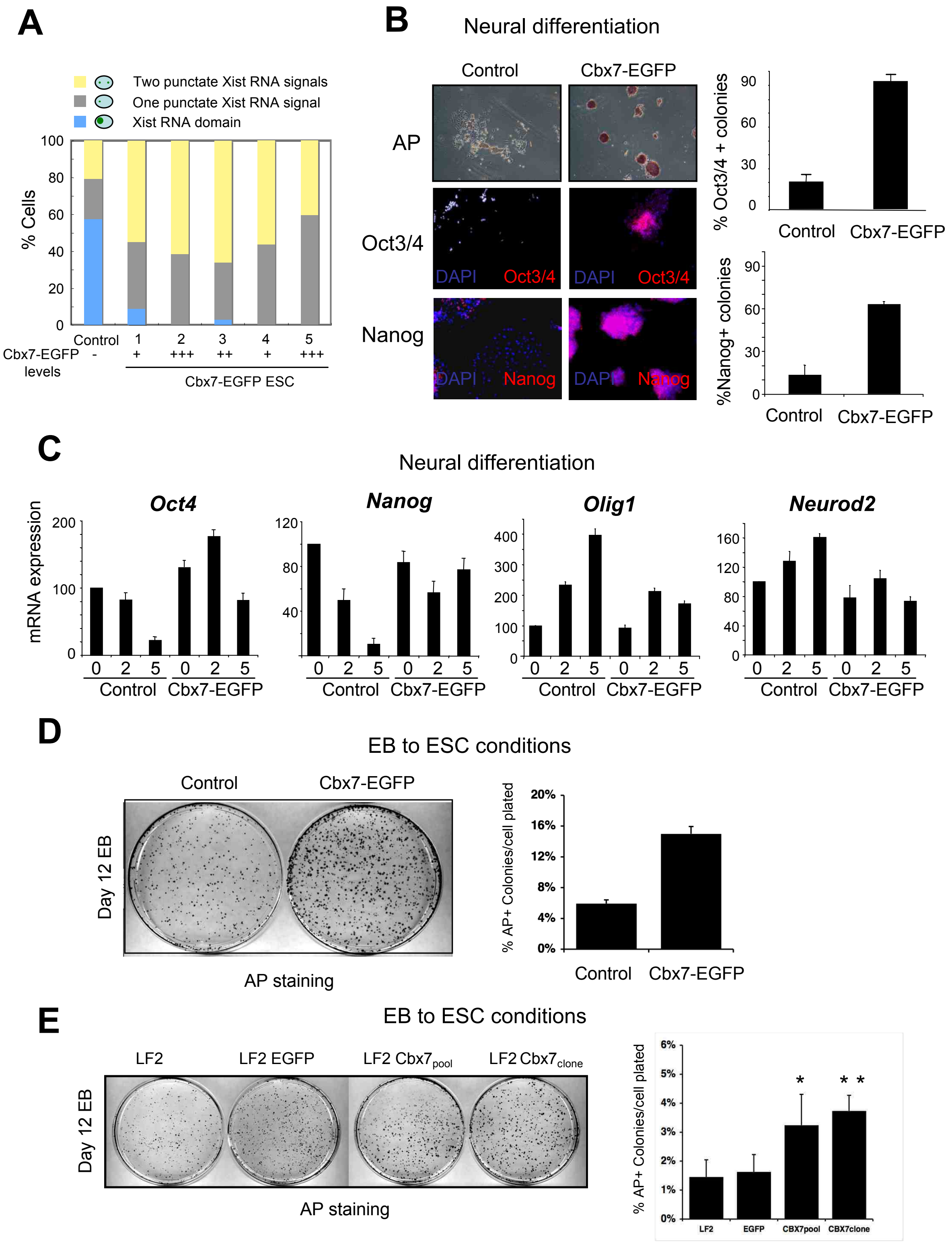

O'Loghlen *et al.* Sup Figure S6

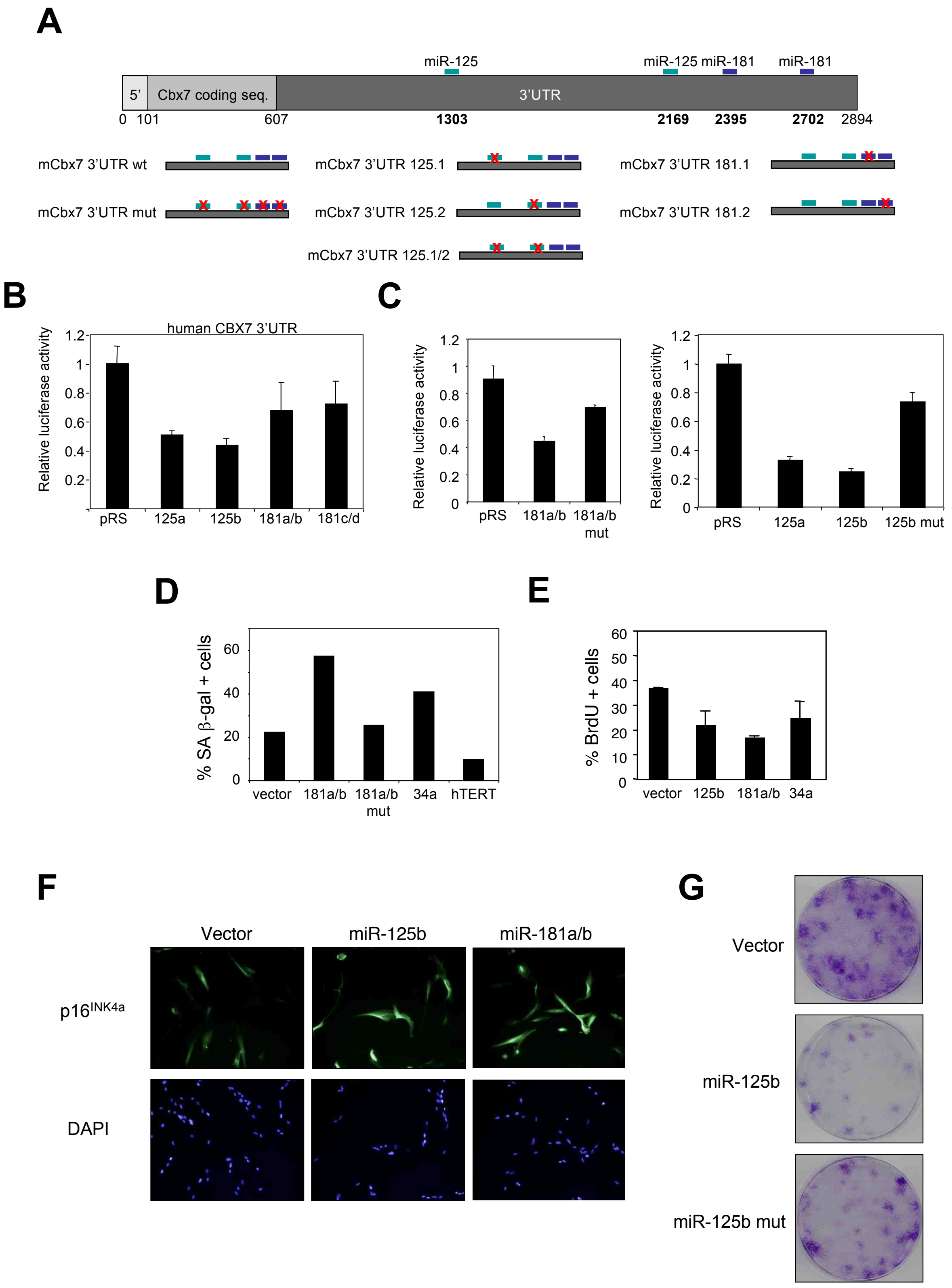

O'Loghlen *et al.* Sup Figure S7

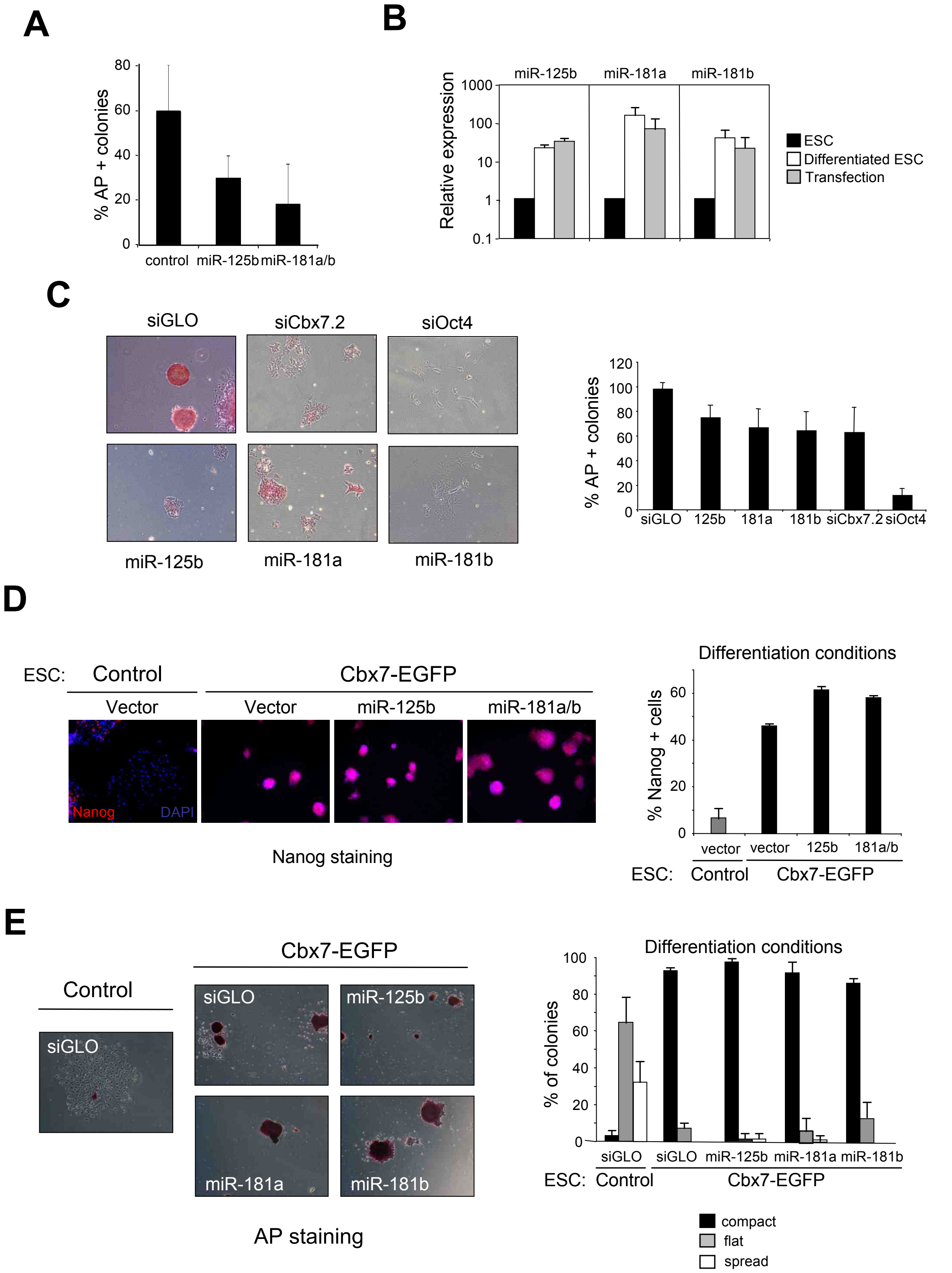

SUPPLEMENTAL FIGURE LEGENDS

Supplemental Figure S1 (related to Fig 1). Cbx7 expression is associated with pluripotency. (A) Cbx7 binds to H3K27me3 in ESC but not in differentiated ESC. H3K27me3 histone peptide pull-downs were performed with nuclear extracts of LF2 ESC prior or after 5 days of RA-induced differentiation. Biotinylated histone peptides (H3 peptide, residues 18 to 37, unmodified or trimethylated at K27) were conjugated to Streptavidin beads, and peptide pulldowns followed by Cbx7 IB were conducted as described in the Sup. Methods. Streptavidin served as loading control for the pulldown. I, input; U, unmethylated; me3, H3K27me3 peptide. (B) Ratio of expression of Cbx proteins in ESC versus NSC. The expression of the Pc homologs was monitored in ESC or neural stem cells (NSC) by qRT-PCR, relative expression is shown. Cbx7 expression is higher in ESC. (C-E) Expression of Cbx2, Cbx4, Cbx6 and Cbx8 during differentiation. (C) Expression during embryoid body (EB) differentiation of 46C ESC analyzed by qRT-PCR. (D) Expression during neural differentiation of Oct4-GIP ESC was analyzed by qRT-PCR. (E) Expression during RA-mediated differentiation of P19 teratocarcinoma cells was analyzed by qRT-PCR.

Supplemental Figure S2 (related to Fig 2). Expression of PRC1 components during ESC differentiation and in other cell types. (A) PGK12.1 ESCs expressing Cbx7-EGFP and control cells were generated as described under experimental procedures and probed for levels of endogenous and exogenous Cbx7 protein, using anti-GFP or anti-Cbx7 antibodies. These cells were used to identify Cbx7-containing PRC1 complexes in ESCs as described in Fig 2B. (B) Expression of PRC1 components in ESC versus NSC. The expression of the PRC1 components was monitored in ESC or NSC by qRT-PCR and normalized to ESC. (C-D) Expression of PRC1 components during differentiation. (C) Expression during neural differentiation of Oct4-GIP ESC was analyzed by qRT-PCR. (D)

Expression during RA-mediated differentiation of P19 teratocarcinoma cells was analyzed by qRT-PCR.

Supplemental Figure S3 (related to Fig 3). Dynamic interplay between Cbx7 and Cbx8 expression during differentiation. (A) Female (LF2) and male (E14, CCE) mESCs were differentiated with RA for 0, 2 and 4d and the levels of Oct4, Cbx7, Cbx8 and histone H3 in the chromatin fraction were assessed by IB. Amido black staining was included as an additional loading control. (B) ChIP showing binding of Cbx7 and Cbx8 at the *Sox3* and *Nr2f2* loci in ESC, differentiated ESC (d7), NSCs and MEFs. Similar trends were observed with multiple primer sets for each locus (data not shown, primer sets listed in Sup. Information). (C) Analysis of genome-wide ChIP data from (Marson et al., 2008) shows Nanog, Oct4 and Sox2 binding upstream of *Cbx7* in ESCs.

Supplemental Figure S4 (related to Fig 4). Cbx7 levels contribute to maintenance of pluripotency in ESCs. (A) A schematic representation of the mouse Cbx7 mRNA, indicating the target sites for siRNAs and lentiviral shRNA vectors used in this study. (B) The effect of 2 lentiviral shRNA vectors (shCbx7.1 and shCbx7.2) in silencing Cbx7 expression was assessed by IB. (C) ESC were infected with an empty vector (vector) or lentiviral shRNAs targeting Cbx7 (pLKO-shCbx7.1 and pLKO-shCbx7.2). Percentage of AP-positive colonies with a compact, flat or spread morphology are shown, along with representative pictures. (D) $Cbx7^{+/+}$ or $Cbx7^{+/-}$ ESC (clones B05 and F05) were infected with a control vector (vec.) or a lentivirus expressing Flag-Cbx7 (F-C7). IB was performed using antibodies against Cbx7 (left). F, indicates a doublet corresponding to Flag-Cbx7. C, indicates a band corresponding to endogenous Cbx7. Percentage of AP-positive colonies that showed a compact, flat or spread morphology in the experiments are shown (right). (E) Representative pictures of AP-stained colonies of the experiment described in (D). Note that this experiment was conducted using feeder cells. (F) The expression of Nanog is not affected after depletion of Cbx7 using siRNA in ESCs.

Supplemental Figure S5 (related to Fig 5). Cbx7 expression blocks ESC differentiation and X inactivation. (A) PGK12.1 mouse female ESC lines stably expressing Cbx7-EGFP were generated. 5 clones (1-5) with different levels of Cbx7-EGFP expression are shown here. Cbx7-EGFP expression level was determined by immunoblotting with anti-Cbx7 antibodies (data not shown). Following 5 days of RA treatment to induce differentiation, Xist RNA was detected by RNA FISH (n > 100 cells for each clone). We used clone number 5 for the experiments presented in the paper. This panel relates to Figure 5B in which the quantification of control ESC and clone 5 of the Cbx7-EGFP ESC are shown. (B) Control and Cbx7-EGFP ESC were subjected to neural monolayer differentiation for 4 days and stained with AP or subjected to Oct3/4 or Nanog IF. Representative images were taken of AP staining (top), Oct3/4 IF (middle) or Nanog IF (bottom). The percentage of Oct3/4 positive colonies and Nanog positive colonies are represented. This panel relates to Fig 5C. (C) Control and Cbx7-EGFP ESCs were subjected to neural monolayer differentiation and RNA was extracted at different points. The expression of Oct4, Nanog, Olig1 and Neurod2 was analyzed by qRT-PCR. (D) Control ESC or Cbx7-EGFP ESC were cultured in non-attachment conditions without LIF to form EBs. EBs were then dissociated and plated back in ESC medium at day 12. Number of ESC-like colonies was analyzed by AP staining after 5 days. Percentage of AP positive colonies formed per cells plated is represented. This panel relates to Fig 5D, which shows a similar experiment at day 20. (E) LF2 cells were cultured and analyzed as described in (D). Representative images and the percentage of AP positive colonies formed per cells plated are shown. * $p<0.005$, ** $p<0.00005$. This panel relates to Fig 5F, which shows a similar experiment at day 20.

Supplemental Figure S6 (related to Fig 6). Regulation of Cbx7 by miRNAs of the miR-125 and miR-181 families. (A) A schematic representation of the mouse Cbx7 mRNA,

indicating the target sites for microRNAs of the miR-181 and miR-125 families, and the battery of reporter constructs used to reveal the functional target sites on Cbx7 3'UTR. (B) A luciferase reporter assay identifies miRNAs from the miR-125 and miR-181 families as regulators of human CBX7 3'UTR. (C) A luciferase reporter assay shows that mutant versions of miR-125 and miR-181 families cannot downregulate a mouse Cbx7 3'UTR reporter. (D, E) Infection of IMR90 cells with vectors expressing miR-181a and miR-181b, miR-125b and a vector expressing seed mutant versions of the miR181a/b (mut). SA-β-Gal activity (D) and BrdU incorporation (E) were measured. miR-34 has been previously shown to induce senescence in IMR90, or apoptosis in other cell types (He et al., 2007) and was used as a control in these assays. (F) IMR90 cells expressing miR-125b or miR181a/b present elevated expression of $p16^{INK4a}$ as assessed by IF. (G) Growth arrest of IMR90 cells is induced by miR-125b but not by a seed-specific mutant version as shown by crystal violet staining.

Supplemental Figure S7 (related to Fig 7). Expression of miR-125b or miR-181a/b accelerates ESC differentiation. (A) Expression of miR-125b or miR-181a/b by retroviral infection causes a loss of ESC properties. Quantification of AP positive colonies is shown. (B) ESCs were transfected with miR-125b, miR-181a or miR-181b synthetic miRNA mimics and the relative expression levels, compared to those of ESC and differentiated ESC (day 6) were measured using Taqman probes. (C) Expression of miR-125b, miR-181a or miR-181b causes a loss of ESC properties. 3 days after transfection with miRNA mimics, pluripotency was examined by AP staining. Representative images and the percentage of AP positive colonies are shown. (D) Control ESC or ESC expressing Cbx7-EGFP (which lacks a 3'UTR, and therefore is resistant to miRNA downregulation) were infected with a control vector or vectors expressing miR-125b or miR-181a/b. Cells were switched to differentiation conditions and after 4 days cells were subjected to Nanog IF. Representative images and quantification of Nanog positive cells are shown. (E) Control ESC or ESC expressing Cbx7-EGFP (lacking a 3'UTR) were transfected with siGLO or synthetic mimics

for miR-125b, miR-181a and miR-181b. Cells were switched to differentiation media and after 4 days, stained with AP. Representative pictures and quantification of colonies with different morphology are shown.

SUPPLEMENTAL EXPERIMENTAL PROCEDURES

Plasmids The miR-181a/b and miR-125b seed mutants were generated by mutagenesis changing 5 bp (miR-181a) or 6 bp (miR181b and miR-125b) in the seed region. psiCHECK2-Cbx7-3'UTR was prepared by subcloning Cbx7 3'UTR from the full length mouse Cbx7 cDNA (Gil et al., 2004) by PCR after the 3' end of the *Renilla* luciferase gene. The human CBX7-3'UTR reporter was cloned likewise using a fosmid construct as template. psiCHECK2-Cbx7-3'UTR mutant constructs were created by mutagenesis deleting the 6-bp miRNA recognition region. Lentiviral pLKO-based shRNA targeting Cbx7 (pLKO-shCbx7.1 and pLKO-shCbx7.2) were obtained from Thermo Scientific (clone IDs were TRCN0000096729 and TRCN0000019145 respectively). To generate Cbx7-EGFP, mouse Cbx7 was cloned into pCAGGS-L/EGFP vector and cotransfected with p10L7 HygroR vector into PGK12.1 cells to derive PGK12.1. Cbx7-EGFP ESCs. To generate Cbx7-Flag-HA, mouse Cbx7 was first cloned in pOZ-FH-C vector for 3' Flag-HA tag addition. 3' Flag-HA tagged mouse Cbx7 was subsequently cloned into pTrip vector. A plasmid encoding Cbx7-Flag was generated by cloning Cbx7-Flag in the BamHI site of the VIRSP lentiviral vector.

Antibodies. The following antibodies were used in this study: anti-BrdU (A21303, Invitrogen), mouse monoclonal anti-p16^{INK4a} (JC-8, CRUK), mouse monoclonal anti-anti-p53 (sc-126, DO1, Santa Cruz Biotechnology), mouse anti-Ring1b (kindly provided by Haruhiko Koseki, RIKEN Research Center for Allergy and Immunology, Yokohama), anti-β-Actin (sc-47778, Santa Cruz Biotechnology or A5441, Sigma), rabbit anti-Cbx8 (Bethyl Laboratories), rabbit anti-Cbx8 (kindly provided by Adrian Bracken, Trinity College Dublin), rabbit anti-Cbx7 (ab21873, Abcam), rabbit anti-Cbx7 (07-981, Millipore), rabbit monoclonal H3 (CT-PAN 05-928, Millipore), mouse anti-Nanog (ab80892, Abcam), anti-Nanog (REC-RCAB0002P-F,Cosmo Bio), anti-Oct4 (sc8628; SantaCruz), mouse monoclonal anti-Oct4 (611202, BD Transduction Labs or sc-5279, Santa Cruz Biotechnology), anti-Sox2 (sc17320x; SantaCruz), anti-H3K4me3 (ab1012, Abcam), anti-H3K27me3 (07-449,

Millipore) and control antibody IgG (12-370, Millipore). Anti-Cbx6 (MRO19) antibodies were raised in rabbit (Cancer Research UK).

Cell culture and differentiation assays. Mouse ESC lines B05 ($Cbx7^{+/-}$) and F05 ($Cbx7^{+/-}$) were generated by the Wellcome Trust Sanger Institute's knockout mouse project (http://www.komp. org) production center (W.C. Skarnes, et al., manuscript in preparation). The Cbx7 heterozygous mutant 'KO first' allele contains a standard β-geo trapping cassette targeted by replacement mutagenesis into the intron 1-2 of Cbx7. 46C (Aubert et al., 2003) and Oct4-GIP reporter (Ying et al., 2002) ESCs were maintained in KO-DMEM supplemented with 10% FBS, L-Glutamine 2mM, Non Essential Aminoacids (NEAA) 100 µM, ß-Mercaptoethanol 0.1 mM and LIF 1000 U/ml. P19 mouse teratocarcinoma cells were maintained in αMEM media supplemented with 10% FBS. Mouse ESC lines B05 ($Cbx7^{+/-}$) and F05 ($Cbx7^{+/-}$), CCE, E14, ZHBTc4, LF2, and PGK12.1 were plated on 0.1% gelatin-coated plates or on a feeder layer of irradiated MEFs, and maintained in DMEM supplemented with 15% FBS, 1 mM L-glutamine, 0.1 mM nonessential amino acids, 100 µg ml−1 penicillin, 100 µg ml−1 streptomycin, 1 mM sodium pyruvate, 0.1 mM 2-mercaptoethanol and recombinant LIF (Millipore). Retinoic acid induced differentiation was performed by plating ESC on 0.1% gelatin-coated plates in ESC media lacking LIF with Retinoic acid (10^{-7}M). Embryoid body (EB) differentiation was performed by plating ESCs into non-adherent conditions in ES media lacking LIF for up to 20 days. ZHBTc4 were differentiated through the addition of doxycycline as previously described (Niwa et al., 2000). For neural differentiation, ESC were cultured as described (Ying et al., 2003). P19 mouse teratocarcinoma cells were differentiated using 1 µM of All-trans-Retinoic acid (Sigma). HEK293T and IMR90 cells were obtained from the ATCC and maintained in Dulbecco's modified Eagle's medium (Invitrogen) with 10% fetal bovine serum (PAA) and 1% antibiotic-antimycotic solution (Invitrogen).

Mass Spectrometry. SILAC-labelled total or nuclear extracts from ESC, differentiated ESC, Cbx7-EGFP ESC and MEF were generated as described (Graumann et al., 2008)

and used for peptide pull-down, Ring1b IP or GFP IP. Quantitative mass spectrometry experiments were performed essentially as described (Vermeulen et al., 2010). The 3D mass spectrum presented in Fig 1A was generated using MaxQuant (Cox and Mann, 2008).

Peptide pulldown assays. Peptide pulldown assays were performed as described (Wysocka, 2006) with small modifications. Biotinylated histone peptides corresponding to aa 18-35, 18-37 or 18-38 of histone H3, unmodified (U) or trimethylated on K27 (me3) were used. Peptides were conjugated to avidin agarose beads (Pierce, 20219) or Neutravidin resin for 4 hrs at RT. Peptide pulldown assays were performed using a solubilised chromatin fraction, which was incubated for 3 hours at 4 C. To prepare the extracts, nuclei were isolated by incubation in ice-cold sucrose buffer (0.32M sucrose, 10mM Tris pH 8.0, 3mM $CaCl_2$ 2mM MgOAc, 0.1mM EDTA and 1mM DTT) supplemented with proteinase inhibitors and either 0.5% or 1% TritonX-100. The quality of the nuclear preps was confirmed visually. 0.2-1x10^8 nuclei were used per assay. The nuclei were incubated in lysis buffer (20Mm HEPES pH 7.9, 25% glycerol, 420 mM KCl, 1.5 mM $MgCl_2$, 0.2mM EDTA, 1mM DTT) and subjected to 3 cycles of freezing and thawing, followed by centrifugation. The supernatant was collected as the nuclear fraction, while chromatin pellets were solubilised by MNaseI digestion. The nuclear and chromatin fractions were pooled together and used for the chromatin capture assay. For each assay, 3 independent extractions were performed and the extracts were pooled together. Extracts were adjusted to 150 mM KCl and pre-cleared. Pre-cleared extracts were then incubated overnight with C-terminally biotinylated histone peptides coupled to Neutravidin resin or avidin agarose beads. Beads were then washed 4 or 5 times with ice-cold wash buffer (20mM HEPES pH 7.9, 20% glycerol, 150mM KCl, 0.2mM EDTA, 0.2% TritonX-100). Bound proteins were eluted by boiling in Laemmli buffer and loaded on SDS-PAGE gel and detected by mass-spectrometry or immunoblotting. Streptavidin blotting served as a loading control for peptides in the immunoblot experiments

GFP immunoprecipitation. GFP-trap beads (Chromotek) were used to precipitate GFP from ESC expressing GFP or Cbx7-EGFP. Approximately 4 mg of total cell lysate were used per pulldown in a buffer containing 150mM NaCl, 1% NP40, 50mM Tris-HCl pH 7.6 and protease inhibitors. After incubating samples at 4°C for 2 hours, beads were washed repeatedly and eluted with 2x Laemmli buffer at 95°C for 10 min.

Ring1b immunoprecipitation. Approximately, $2.5x10^8$ cells were used per IP. Nuclear extracts prepared as described for the peptide pulldown assays were precleared with 100 μl of protein G-agarose for 1 h at 4°C, prior to overnight incubation at 4°C with Ring1B antibody (using 5 μg antibody/mg extract). In the morning, Protein G-agarose beads were added and incubated for 3 additional hours at 4°C. After incubation, beads were washed several times and eluted with 2x Laemmli buffer at 95°C for 10 min.

Senescence-associated β-Galactosidase staining. IMR90 fibroblasts (5×10^4) were seeded in 6-well plates. Two days later, the cells were fixed with 0.5% glutaraldehyde (*w*/*v*) for 15 min and then washed twice with 1 mM $MgCl_2$ in PBS (pH 6.0). X-Gal staining solution (1 mg mL^{-1} X-Gal, 5 mM $K_3[Fe(CN)_6]$ and 5 mM $K_4[Fe(CN)_6$ in 1 mM $MgCl_2$/PBS (pH 6.0)) was added to the cells for 2–24 h, after which the cells were washed with water and stored at 4 °C, in the dark. The cell nuclei were stained with 4',6-diamidino-2-phenylindole (DAPI) for 30 min. Bright field and DAPI images were taken and the percentage of SA-β-Gal-positive cells was determined upon counting of at least 100 cells per condition.

Quantitative RT-PCR analysis. Total RNA was extracted using miRCURY RNA isolation kit (Exiqon) or RNeasy kit (Qiagen). cDNAs were generated using SuperScript II reverse transcriptase (Invitrogen). For miRNA reverse transcription TaqMan miRNA reverse transcription kit was used with specific miRNA primers. PCR reactions were performed in an Opticon 2 Real-Time PCR Detection System (BioRad) using Power SYBR Green Master Mix or TaqMan Universal PCR Master Mix (Applied Biosystems). Expression was normalized to ribosomal protein S14 (RPS14), ribosomal protein L7 gene or U6 snRNA for

mouse miRNAs and RNU6B for human miRNAs. A list of primers and Taqman probes used is presented as a table in the Sup. Experimental Procedures.

Chromatin immunoprecipitation (ChIP). For ChIP analysis proteins bound to DNA were cross-linked using 1.42% formaldehyde for 10 min at room temperature followed by addition of glycine (0.125 M final concentration) for 5 min at room temperature. After washing with ice-cold PBS, cells were collected by centrifugation, washed once with ChIP buffer (150 mM NaCl, 50 mM Tris-HCl (pH 7.5), 5 mM EDTA, NP-40 (0.5% vol/vol), Triton X-100 (1.0% vol/vol) and Complete protease inhibitors, Roche), and lysed in the same buffer for 30 min on ice. Following sonication protein-DNA complexes were immunoprecipitated overnight at 4°C with rotation using primary antibodies Immunoprecipitated chromatin was incubated with Protein G beads for another 5 h at 4ºC and then washed and eluted by incubation with elution buffer (2% SDS, 100 mM NaHCO3, 10 mM DTT). Crosslinking was reversed by incubation at 65˚C overnight. DNA was purified by phenol–chloroform extraction, followed by ethanol precipitation. Real-time PCR reactions using SYBR green (Roche) were performed in triplicate using primers for the correspondent genomic regions. Fold enrichment over 1% input was calculated using the 2DeltaCt method. The data presented in Fig 3B and Sup Fig S3B was performed with a primer set per gene, but similar ChIP using 4 different sets of primers close to the TSS of each gene was also performed obtaining similar results. Primers for the experiments shown in Fig 3C and Fig 3E represent genomic regions of Cbx7 and Cbx8 around the TSS (0). The coordinates for the positive control region (NOS), which is bound by all three ES cells regulators Nanog, Oct4 and Sox2, are located 200bp upstream of the Nanog gene, chr6:123352993–123353158 (mm5 genome build). The primer sets used for these experiments are listed as a table at the end of the Sup. Experimental Procedures.

Bioinformatic analysis. Raw ChIP-seq data pluripotency factors binding (Nanog, Sox2 and Oct4) (Marson et al., 2008) were downloaded from GEO (GSE11724), with data in SRA format transformed into Fastq format using the SRA toolkit 2.0.1. Sequence data was

aligned to MM9 genome using Bowtie version 0.12.7, coverage for aligned reads calculated using Bedtools version 2.12.2 and the resulting bigWig files visualized in the UCSC genome browser.

TABLES FOR SUPPLEMENTAL PROCEDURES

Supplemental Table of qRT-PCR primers and Taqman probes used in this study

List of primers for qPCR

Mouse

Target	Forward primer	Reverse primer
Oct4	CTGTAGGGAGGGCTTCGGGCACTT	CTGAGGGCCAGGCAGGAGCACGAG
Nanog	AGGGTCTGCTACTGAGATGCTCTG	CAACCACTGGTTTTTCTGCCACCG
Cbx7	TGCGGAAGGGCAAAGTTGAAT	ACAAGGCGAGGGTCCAAGA
Rps14	GACCAAGACCCCTGGACCT	CCCCTTTTCTTCGAGTGCTA
Sox2	GCCGAGTGGAAACTTTTGTCG	GCAGCGTGTACTTATCCTTCTT
Sox3	TTCGCACTCGCAGCGCGCGTGCCTC	ACGGCCAAACTTTCGGTCCTC
Fgf4	CTACTGCAACGTGGGCATCG	CGCTGCACCGGAGAGAGC
Fgf5	CTGTATGGACCCACAGGGAGTAAC	GTAGAGGAGGATAACACGCACTG
Flk1	TTTGGCAAATACAACCCTTCAGA	GCAGAAGATACTGTCACCACC
FoxA2	TAGCGGAGGCAAGAAGACC	CTTAGGCCACCTCGCTTGT
Gata4	CCCTACCCAGCCTACATGG	ACATATCGAGATTGGGGTGTCT
Gata6	ACAGCCCACTTCTGTGTTCCC	CTTCTGTTTCCGATCAGCTCCCTTG
NKX2-2	GGTGGAGCGATTGGATAAGA	TGCCATCAACCTTTTCATCA
NKX2-9	CCACACCAAAGTCAGGAGGT	CCGAGTCTTGGAGACAGTCC
HoxA4	GATGAAGAAGATCCACGTGAGCG	CCAGTTCCAAGACTTGCTGCC
L7	AGCGGATTGCCTTGACAGAT	AACTTGAAGGGCCACAGGAA
MixL1	TTGAATTGAACCCTGTTGTCCC	ACTCTAGGTATCCGTCAGGGAAG
Msi-1	CCGGAGTTACACAGGCCTTG	GGGATAGCTGTGAGCTCGGG
Nestin	CCCTGAAGTCGAGGAGCTG	CTGCTGCACCTCTAAGCGA
Neurod2	GCTGCTGAGTCTCGGGATAG	GCTTGGCCTCTCTCTTTCCT
Olig1	TGAATCCCACCTGTTTAGAGCC	CGATGCTCACGGATACGAGAATAG
Pax3	ATAAGCCCAGGACACAGAGTTGTG	CTCGGTCAAGGATGGAAGC
Rex1	CAGTCCAGAATACCAGAGTGGAA	GGTCTTCATGGATTCCTCCAGCTTC
RhoX5	GGCCCAAGCTCAGAATC	CTGAATAGGATCAATGATGAAG
RhoX6	TGAATAGGCTGGCTCAACTGCG	AAAGGGCTCTCCTCATCCGAAACC
RhoX9	TCAAGAAGAGCCTGCTCCAT	CCAGCCTGTGGATTTCTCAT
Irx3	CGCCTCAAGAAGGAGAACAAGA	CGCTCGCTCCCATAAGCAT
Math1	AGATCTACATCAACGCTCTGTC	ACTGGCCTCATCAGAGTCACTG
Dach1	ACATGCACATACGCACACTTT	AAGAGGTCAAGACAGGAACATCA
Brachyury	CCGGTGCTGAAGGTAAATGT	CCTCCATTGAGCTTGTTGGT
Goosecoid	CTCGGAGGAGTCAGAAAACG	CAGTCCTGGGCCTGTACATT
Gata6	GAGCTGGTGCTACCAAGAGG	TGCAAAAGCCCATCTCTTCT
Sox1	TGAACGCCTTCATGGTGTGGTC	GCGCGGCCGGTACTTGTAAT

Human

Target	Forward primer	Reverse primer
CBX7	GGATGGCCCCCAAAGTACAG	TATACCCCGATGCTCGGTCTC
p16	CGGTCGGAGGCCGATCCAG	GCGCCGTGGAGCAGCAGCAGCT
RPS14	TCACCGCCCTACACATCAAACT	CTGCGAGTGCTGTCAGAGG

List of TaqMAN probes

Target (mouse)	Applied Biosystems Reference
Cbx2	Mm00483084_m1
Cbx4	Mm00483089_m1
Cbx6	Mm00503891_m1
Cbx7	Mm00520005_m1
Cbx8	Mm00489229_m1
TBP	Mm00446971_m1
miR-125a	448
miR-125b	449
miR-181a	480
miR-181b	1098
U6 snRNA mouse	1973
RNU6B human	1093

Supplemental Table including siRNAs used in this study

List of siRNA

Target	Targeting sequence
siCbx7.2	AACCACCTGCCTATCTCTTAA
siCbx7.3	TGGGACTAAATGCCTGTACAA
siOct4.1	CCCGGAAGAGAAAGCGAACTA
siNanog.10	CAGGTTAAGACCTGGTTTCAA

Supplemental Table including primer sets used for ChIP analysis in this study

Gene	Forward primer	Reverse primer
**Gata4**		
PS1	GCGATGGCCTCTGAAATCCTCAGAAT	CCCCAATCCTAAACAATGCCAGTCAG
PS2	GGAAGGAACTACAGTCCTTGAATGGG	GGAAGGAACTACAGTCCTTGAATGGG
PS3	TTTGCCTGGAATAGGGAGGTAGGTAG	TAGGCCAAAGTCAAAAGCGGAGCAGT
PS4	GGAACTTGGGATGAACGTTGTAGGGT	GCCTGGCCTAGACTTTTCTCTCTCTT
**Neurog2**		
PS1	CATTTGTCCTCTCTCGCCACTTGT	TTGTGATGGCCCAAAGCCCTAAAG
PS2	CAGATGACACTCAGGACTCACTCACT	AATTGCTGTCTGTCCCTCCCCTTT
PS3	GGGGAGAGGGACTAAAGAAAGGGAAA	GCTCTCCCTCCCCAGCTTATTCTTTT
PS4	CTCGACCTTTTAGGGGAGAAGGAACT	AGGAAACACGTGTGTGGCTGAT
**NR2F2**		
PS1	AAGCGGTTTGGGACCTTGAACA	CCCACACAGCACAAGGAGAAAAGT
PS2	GCGCCTATAAATCGCATTCCCT	CGCGGAGAGAAAAGAGCAGAATCA
PS3	ACGTTCTGCTCCCACTCTCTTT	TTTGTTTCCCTCGGCTCAGCTT
PS4	GCCACTCGTACCTGTCCGGATATATT	TTGTTAGGCTGCATGCACTGAC
**Sox3**		
PS1	CGGCTTACTTGGGTCCTAAAACTTGG	CCTGAGGCTGTTGCTAAGTGAAGAG
PS2	CCATCAATTCCAAGGAAGGCTTCTCAG	GCTTGGGACGTTGAGTGTATAACGGA
PS3	CCGGATCTGAGCAGGTATATAAGGGA	GCTTCTCTCACCTGATGCGTTCTCT
PS4	TCCTCTTCCCGGATTCCTAGGTTCTT	GGGCAACCTCACTCAGTTCTCGATTA
**Cbx7**		
PS3	GCCACTTCATTTCGGACCTTCTCA	GTCTAAGGTCCATCCCATCTCTCAGTTC
**Cbx8**		
PS2	AAAAGGGCAATGTGGGGATAGCTG	GACCTTTTGCTCCTAGGCTCTCTTTC
**Cbx7**		
-3.0Kb	GGATTCCTGTGGTTTTGTGTG	ACACAAAGGAAGCCCCTTCTA
+0Kb	ACCTTAGACTGCCAGGAGCA	ACCCCCTAGTGTTCCCTCAC
+3.0Kb	TTGGCTTGTGAGTGATCTGC	CAATACCCAGGTCACCATCC
**Cbx8**		
-3.0Kb	AAGTACTAAGCGCGGGGACT	GGGTCATCAACTGCTGGTTT
+0Kb	CTACGCAGGGAGCCCTAATA	GTCCACTCCAGAGACCCAGA
+3.0Kb	ATCTCCCCGTTACCTCGATT	AAACAACTGTCTGGCGCTCT

SUPPLEMENTAL REFERENCES

Aubert, J., Stavridis, M.P., Tweedie, S., O'Reilly, M., Vierlinger, K., Li, M., Ghazal, P., Pratt, T., Mason, J.O., Roy, D., *et al.* (2003). Screening for mammalian neural genes via fluorescence-activated cell sorter purification of neural precursors from Sox1-gfp knock-in mice. Proc Natl Acad Sci U S A *100 Suppl 1*, 11836-11841.

Cox, J., and Mann, M. (2008). MaxQuant enables high peptide identification rates, individualized p.p.b.-range mass accuracies and proteome-wide protein quantification. Nat Biotechnol *26*, 1367-1372.

Graumann, J., Hubner, N.C., Kim, J.B., Ko, K., Moser, M., Kumar, C., Cox, J., Scholer, H., and Mann, M. (2008). Stable isotope labeling by amino acids in cell culture (SILAC) and proteome quantitation of mouse embryonic stem cells to a depth of 5,111 proteins. Mol Cell Proteomics *7*, 672-683.

He, L., He, X., Lim, L.P., de Stanchina, E., Xuan, Z., Liang, Y., Xue, W., Zender, L., Magnus, J., Ridzon, D., *et al.* (2007). A microRNA component of the p53 tumour suppressor network. Nature *447*, 1130-1134.

Niwa, H., Miyazaki, J., and Smith, A.G. (2000). Quantitative expression of Oct-3/4 defines differentiation, dedifferentiation or self-renewal of ES cells. Nat Genet *24*, 372-376.

Wysocka, J. (2006). Identifying novel proteins recognizing histone modifications using peptide pull-down assay. Methods *40*, 339-343.

Ying, Q.L., Nichols, J., Evans, E.P., and Smith, A.G. (2002). Changing potency by spontaneous fusion. Nature *416*, 545-548.

Ying, Q.L., Stavridis, M., Griffiths, D., Li, M., and Smith, A. (2003). Conversion of embryonic stem cells into neuroectodermal precursors in adherent monoculture. Nat Biotechnol *21*, 183-186.
